# Automatic *online* spike sorting with singular value decomposition and fuzzy C-mean clustering

**DOI:** 10.1186/1471-2202-13-96

**Published:** 2012-08-08

**Authors:** Andriy Oliynyk, Claudio Bonifazzi, Fernando Montani, Luciano Fadiga

**Affiliations:** 1 Section of Human Physiology, Department of Biomedical Sciences and Advanced Therapies, Faculty of Medicine, University of Ferrara, Via Fossato di Mortara 17/19, 44121, Ferrara, Italy; 2 Instituto de Física La Plata (IFLP), CC 67, Calles 49 y 115, 1900, La Plata, Argentina; 3 Normal Physiology Department, Odessa Medical University, 2 Valihovsky lane, 65026, Odessa, Ukraine; 4 The Italian Institute of Technology, Department of Robotics, Brain and Cognitive Sciences, via Morego 30, 16163, Genova, Italy

## Abstract

**Background:**

Understanding how neurons contribute to perception, motor functions and cognition requires the reliable detection of spiking activity of individual neurons during a number of different experimental conditions. An important problem in computational neuroscience is thus to develop algorithms to automatically detect and sort the spiking activity of individual neurons from extracellular recordings. While many algorithms for spike sorting exist, the problem of accurate and fast *online* sorting still remains a challenging issue.

**Results:**

Here we present a novel software tool, called FSPS (Fuzzy SPike Sorting), which is designed to optimize: (i) fast and accurate detection, (ii) *offline* sorting and (iii) *online* classification of neuronal spikes with very limited or null human intervention. The method is based on a combination of Singular Value Decomposition for fast and highly accurate pre-processing of spike shapes, unsupervised Fuzzy C-mean, high-resolution alignment of extracted spike waveforms, optimal selection of the number of features to retain, automatic identification the number of clusters, and quantitative quality assessment of resulting clusters independent on their size. After being trained on a short testing data stream, the method can reliably perform supervised *online* classification and monitoring of single neuron activity. The generalized procedure has been implemented in our FSPS spike sorting software (available free for non-commercial academic applications at the address: http://www.spikesorting.com) using LabVIEW (National Instruments, USA). We evaluated the performance of our algorithm both on benchmark simulated datasets with different levels of background noise and on real extracellular recordings from premotor cortex of Macaque monkeys. The results of these tests showed an excellent accuracy in discriminating low-amplitude and overlapping spikes under strong background noise. The performance of our method is competitive with respect to other robust spike sorting algorithms.

**Conclusions:**

This new software provides neuroscience laboratories with a new tool for fast and robust *online* classification of single neuron activity. This feature could become crucial in situations when *online* spike detection from multiple electrodes is paramount, such as in human clinical recordings or in brain-computer interfaces.

## Background

Electrophysiological recording of single neuron activity represents a fundamental tool for investigating brain functions. Since a recording electrode often picks-up spikes from more than one neuron, a spike sorting technique is needed to identify and separate spikes of different neurons [1,2]. Most currently available computational procedures provide accurate sorting and classification, but are often highly interactive and time-consuming and require specific experience and subjective judgments. Fast automatic methods are available [3-5], but they are usually not as accurate as the *offline* ones and often suffer from problems such as false match or double match, spike overlap and errors in classification [1,6]. Solving the tradeoff between automation, speed and accuracy of spike sorting is thus a crucial challenge in systems neuroscience.

Here, we aim at contributing to the progress of the field by achieving accurate, fast and fully automated spike sorting. To this purpose, we present a new method (and a software package) based on the Fuzzy C-mean (FCM) classification of spike waveforms in the low-dimensional feature space of Principal Components (PCs). Many currently used *offline* spike sorting algorithms already employ Principal Component Analysis (PCA) as a preprocessing step to compress the dimensionality of the patterns to be clustered [1,7]. However, its practical application for fast and automatic separation of neurons is still limited for many reasons. In fact, it is commonly reported that the performance of the PCA heavily depends on the accuracy of the waveform alignment [8], thus requiring strong computations and human supervision of the clustering results [9]. In addition, eigenvectors accounting for the largest variance of the data are not necessarily providing the best separation of the spike classes [10]. Finally, it is often pointed out that PCA, in its basic configuration, is a static technique, not suitable for monitoring of non-stationary behaviour [11], while *in vivo* single unit activity represents mostly non-stationary system with nontrivial dynamics [12].

**Figure 1:**
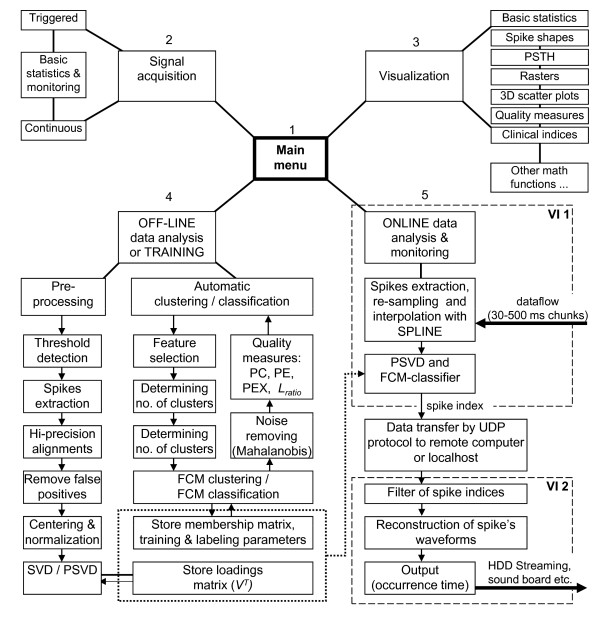
**Functional architecture of the FSPS™ programme.** The main menu (*1*) allows the user to initiate new recordings (*2*), visualize raw data or results (*3*) and to get fast access to any stage of the data processing (*4* and *5*). All other modules of the programme are loop-structured and prompt the user to execute a procedure when necessary. Dynamic links between modules and storage of critical parameters during SVD of data matrix and unsupervised FCM allows the program to solve the invariance problem during PCA, automatically select features to be used in *online* fuzzy classification, choose the best settings for clustering (new or previously calculated for each recording site) and remove noise. Elements included in the dotted block are crucial parameters obtained during multivariate analysis and unsupervised FCM, which are stored externally from the programme for use during fuzzy classification. These are updated when necessary. Output classes (namely, labelled groups of PCs), together with participating elements, thus represent different single neurons of specific action potential waveform, and then indicate their location in the raw signal. Elements marked with dashed line (VI 1 and VI 2) are two separated Virtual Instruments (VI) working simultaneously either on one computer with multi-core processor architecture or two different single-processor computers connected to a LAN. Solid blocks and lines inside VI 1 element – parts of the programme involved in the *online* procedures; dotted blocks and lines – parts that run once to get the prototype, before starting *online* classification.

In this article, we develop and present a spike sorting software called FSPS (Fuzzy SPike Sorting) which is designed to overcome these limitations. The FSPS software (whose architecture is sketched in Figure 1) increases the robustness and speed of PCA-based spike sorting means of several steps. First, it carefully preprocesses the data to improve the alignment of spike shapes. Second, it uses a Partial Single Value Decomposition (PSVD) preprocessing technique to extract PCs [13]. This technique is computationally efficient because it exploits previously computed Single Value Decomposition (SVD) for the further dynamic low-rank approximation of new coming waveforms by means of series of simple matrix operations on the output eigenvectors and at the same times reduces the noise in the components to be sorted [14,15]. Third, the algorithm uses the information obtained during SVD to classify the neuronal waveforms by means of FCM clustering [16-19]. The unsupervised nature of FCM and its ability to detect clusters of different shapes makes it particularly useful for *online* sorting because of its robustness to non-stationary recordings, responsible of the smeared clusters in the high-dimensional feature space. Fourth, to control the accuracy of neuron isolation, the software provides several objective isolation quality measures, including the *L*_*ratio*_ measure which allows a comparison of cross-laboratory clustering [20]. 

The article is organized as follows. First, we describe the spike sorting method used in our approach. We then evaluate its accuracy using simulated spike datasets at different background noise levels proposed by Quiroga et. al. (2004). Then we demonstrate the performance of our method by applying it to the analysis of real extracellular recordings from the macaque premotor cortex, and we investigate the robustness of the algorithm to sample size and inhomogeneities between cluster size to deal with the non-stationarity of the data during the recording session. Finally, we illustrate how our method of spike sorting can be implemented to monitor *online* the activity of single neurons during electrophysiological recordings.

## Implementation

**Figure 2:**
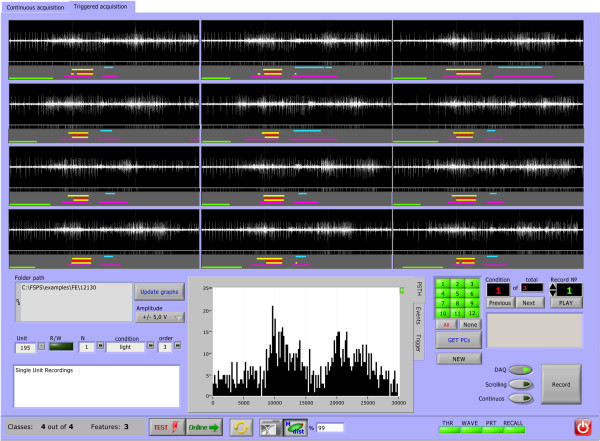
**Interface window for triggered data acquisition and visualization.** An *offline* module of the FSPS programme shows the acquired signal and consents instant acquisition of the two first Principal Components (PC1 and PC2, after full SVD) for each triggered acquisition session. Here we showed the organization of the user-interface window with neural polyspikes acquired during twelve triggered sessions of goal-directed movement in a monkey. Raw data from the electrode and additional digital hardware information were retrieved and visualized (as shown under each session). The resulting histogram of spike occurrence (on the basis of external hardware threshold discriminator) is shown below the trials. The protocol information used later for the analysis is shown to the left of the histogram, while the control buttons to start, stop, overwrite and scroll the trials is shown to its right.

**Figure 3:**
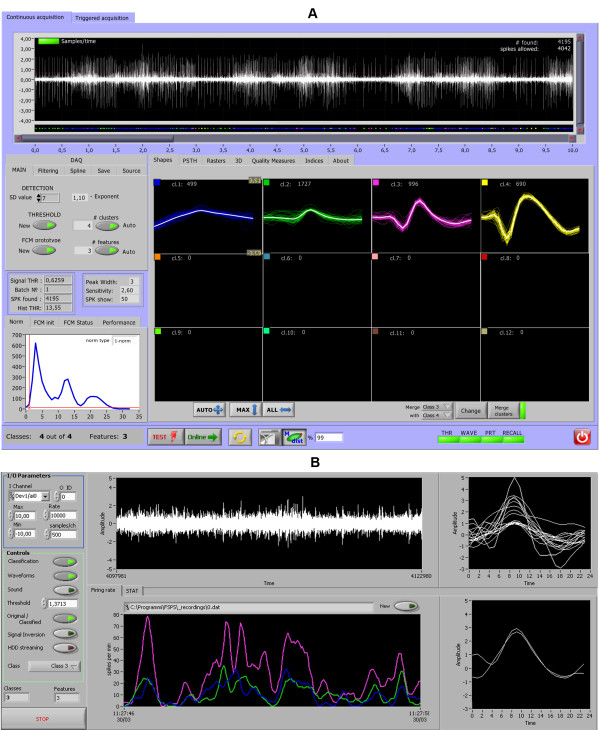
**Fully automated *online *classifier.*A*** - After the period of “TEST” acquisition, which starts once for each recording site, the classification of newly arriving spikes is continual. The control panel on the left-hand side permits some adjustments to automatic thresholding and *spline* interpolation parameters, depending on the digitalization rate of acquisition. Numerical information about the number of spikes and single units, as well as their waveforms, are instantly available to the researcher; ***B*** – interface window of *online* monitoring. This is the most innovative part of our procedure. To ensure good performance we gave the option of sharing most resource-dependent processes, like extraction of PCs and their FCM-classification, between two different processors. In order to achieve this goal we created two separated subprograms (VI 1 and VI 2), running in parallel and linked via UDP protocol, for the transmission of the reduced number of extracted features (PCs) together with a time of spike occurrence, which is reverse-reconstructed and reproduced right after event classification. These two VIs, being processor-dependent, can be run on the same computer when a limited number of recording electrodes is considered. Since the latest versions of LabVIEW (LabVIEW-2009 or higher) can effectively treat multi-core processor architecture and parallel-loop execution, the FSPS software can run on the same computer, sharing the power of multi-core processor (Intel Core i5-2430 M, 2.4 GHz). However, an Ethernet connection may also be useful when experimental conditions necessitate distant *online* monitoring.

The basic strategy of FSPS software is to provide accurate and trustable classification with a minimal supervision and, more importantly, without specific software knowledge (like Python scripting, Matlab toolboxes, C++ etc.). The software supports a large variety of digital acquisition (DAQ) systems (including low-cost ones) and simplifies electrophysiology setup by using the flexible graphical user interface (GUI) of Virtual Instruments (VIs). The spike sorting algorithm was entirely implemented within graphical programming language LabVIEW 2009 (National Instruments, USA), whose DAQ hardware and interfaces became very popular in electrophysiology labs over the last decade. Besides, we choose this software platform for its ability to control the experimental protocol and data acquisition while being able to run the analysis fast and *online* using threaded dataflow methodology [21]. It is also reported that many LabVIEW subroutines shows considerable outperformance when compared to their identical counterpart written in MATLAB (MathWorks, USA) [22]. FSPS high-level schema is sketched in Figure 1. The program allows triggered (Figure 2) and continuous (Figure 3A) acquisition from one or more electrodes simultaneously and offers the user the choice to set all parameters of the analysis automatically or manually, both in case of “test” acquisition and *online* classification (Figure 3B). Besides, the software has the following advanced features: band-pass signal filtering; automatic detection of spikes with evaluation of background noise level and automatic threshold selection; extraction and alignment of spike waveforms; removal of constant DC offset, false positive and noisy spikes; pre-processing with computationally efficient PCA; automatic determination the number of PCs to retain; automatic determination of the number of clusters to be found; offline fuzzy clustering analysis; online fuzzy classification; 2D and 3D visualization tools; quantitative quality assessment of resulting clusters, basic statistics, PSTH, measurements of some clinical parameters of spike trains etc. Additional file 1. The software allows simultaneous visualization/monitoring of activity of several isolated neurons and provides online acoustic feedback about one selected neuron. It has import/export features and allows synchronization of the acquisition with external devices (e.g. digital videorecorders, stimulators etc.). The application is available at http://www.spikesorting.com in the *Download* section. 

### Simulation

Simulated extracellular recordings were used to test the spike sorting procedures. These simulated data were the ones used in [10] and are available *online* at http://vis.caltech.edu/~rodri/Wave_clus/Wave_clus_home.htm. Briefly, simulated signals consisted of spike shapes of three neurons compiled from recordings in the neocortex and basal ganglia. For generating background noise, spikes randomly selected from the database were superimposed at random times and amplitudes. Next, a train of three distinct spike shapes was superimposed on the noise signal at random times. The amplitude of the three spike classes was normalized to have a peak value of 1. The noise level was determined from its standard deviation, which was equal to 0.05, 0.1, 0.15, and 0.2 relative to the amplitude of the spike classes. There were four different example simulations, number from one to four in order of increasing sorting difficulty (see Ref [10]).

### Real multi-unit recordings

Electrophysiological recordings were made from freely behaving, partially restrained, macaque monkey (*Macaca fascicularis*). All experimental protocols were approved by the Veterinarian Animal Care and Use Committee of the University of Ferrara, by the Italian Ministry of Health and complied with the European laws on the use of laboratory animals. The surgical procedure were the same as previously described [23]. Multi-unit recordings were performed by using varnished (Sivamid 595, ELANTAS Deatech S.r.l., Italy) tungsten microelectrodes with impedance 0.15–1.5MΩ (measured at 1 kHz), slowly inserted in the cortex by a hydraulic microdrive (Kopf Instruments, CA, USA; step resolution, 10 μm). Recorded signal was amplified 10,000 times (BAK Electronics, Germantown MD, USA), filtered by a dual variable filter VBF-8 (KEMO Ltd., Backenham, UK) (bandwidth 300–5000 Hz) and digitized (USB-6229, National Instruments, USA) at 10 kHz. During *online* classification, isolated spike shapes of selected unit are flashed on computer display and reproduced by a sound device to provide experimenters with audio feedback on neuron response.

### Spike detection and waveform extraction

**Figure 4:**
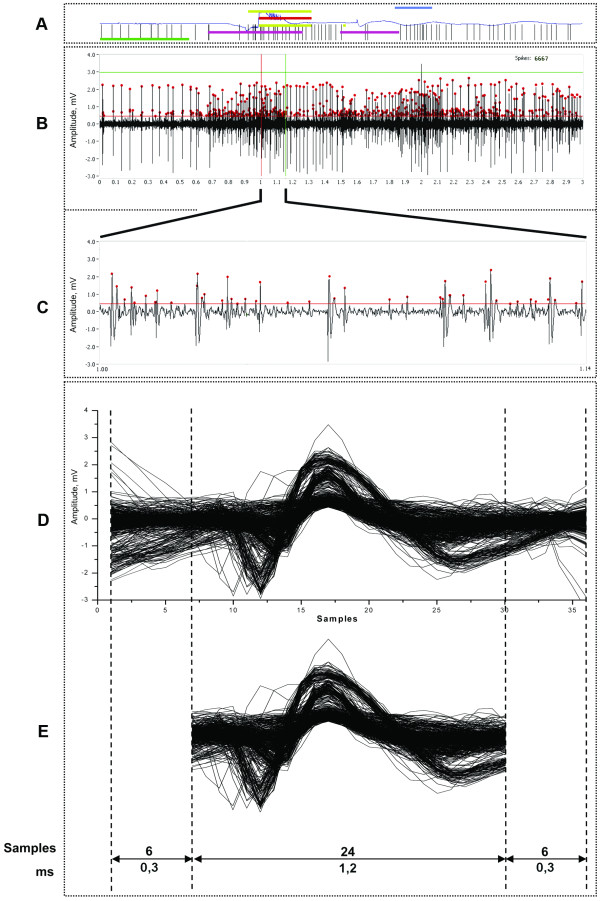
**Visualization of raw signal and collection of spike waveforms.*A*** - information covering each trial of movement execution to monitor the uniformity of trials, where black vertical hatches represent spike occurrence detected using external hardware threshold discriminator; blue line is the infrared signal from the analogue IR-pair; thick coloured horizontal lines are retrieved from the set of digital sensors representing different kinematic parts of executed movement. ***B*** - the visualization of one trial in 3 sec multi-unit recordings, where spikes determined by software discriminator are marked with red dots. ***C*** - an expanding view of the same raw signal, in which the presence of different kinds of spikes is evident; *D* - extracted 1.8 ms of spikes waveforms, aligned to the peak of action potential by *spline* interpolation method; *E* - the same waveforms with 6 truncated samples at the ends, yielded 1.2 ms waveforms to be filled by the data matrix.

The detection of individual spikes in the sampled signal was performed with LabVIEW Peak Detector VI that fits a quadratic polynomial to sequential data points. This algorithm interpolates between sequential data points to find the peak time and reduces errors caused by asynchronous sampling of rapidly changing waveforms, ultimately providing a better alignment of spike shapes. To determine the significance of each peak, the quadratic fit of the peak was tested against the threshold level (*Thr*), automatically adjusted for each recording site [10]:

(1) $$Thr=4\sigma_{\mathrm{noise}};\sigma_{\mathrm{noise}}=median\left\{\frac{\left|x\right|}{0.6745}\right\}$$

where *x* is the bandpass-filtered signal and $\sigma_{\mathrm{noise}}$ is an estimate of the standard deviation of the background noise [24]. Whereas peaks with amplitude lower than the threshold were ignored, peaks higher than threshold were considered for further analysis as follows. Once a significant peak was detected, the whole waveform was collected (eight samples before the peak and ten samples after it, which with our sampling frequency resulted in a total duration of 1.8 ms) and was then interpolated twice to obtain 36 samples for each waveform with cubic *spline* interpolation method [25]. Six samples at the beginning and at the end of each interpolated shape were then removed, thus leading to 24 sample waveforms (1.2 ms, see Figure 4). These parameters were empirically found to be a good compromise between sampling as many points as possible to record all the important phases of action potential, and keeping the number of spike parameters compact to facilitate further analysis. A *n×24* indexed array was then filled with these peak data, rejecting spikes that violate a minimum refractory period after the preceding threshold crossing in order to reduce false positives (see Additional file 2). 

### Extraction of Principal Components

Spike extraction as described above gave origin to a real-valued data matrix *A*^(n×24)^ containing *n* rows to-be-analyzed spike shapes. Before performing further analysis, we centered the collected waveforms by subtracting the row average from every element in a row, which is referred to as *centering across the second mode*[26] in order to remove constant terms in the data. It can be expressed as:

(2) $$X=A-n1^{\text{T}}\text{,}$$

where *A* is a real-valued data matrix *A*^(n×24)^ containing spike shapes, **n** is a vector holding *n*^th^ row average in the *n*^th^ element, **1** is a *n*-vector of ones and *X* is a matrix holding the centering data.

We then extracted the PCs of the spike waveforms using Singular Value Decomposition (SVD). SVD is a factorization approach of a given matrix, and constitutes a powerful computational tool commonly used in many engineering and biomedical applications [27,28]. SVD is analytically presented in standard textbooks on linear algebra and multivariate statistics (see [29] for an extensive review of the method). Let’s consider the matrix *X* to be of rank *r*, in which the rank refers to the maximum number of linearly independent row vectors. This factorization approach captures the most important properties of the matrix *X*, as it allows to decompose the matrix *X* into the product of three matrices:

(3) $$X=USV^{\text{T}}\text{,}$$

where *U* is a *n × 24* left orthogonal matrix of singular vectors giving the principal component *scores* which represent the spike waveforms in term of the PCs; *V* is a *24 × 24* orthogonal matrix detailing the spike profile and mapping the vector space; and *S* is a *24 × 24* diagonal matrix where the diagonal elements are the *singular values* of *X*.

**Figure 5:**
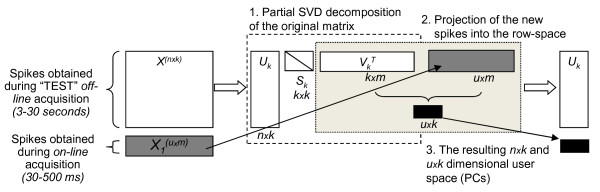
**Schematic diagram of the Partial SVD application for extraction of PCs.** White elements represent standard Partial SVD decomposition used in *offline* or “TEST” clustering. Grey elements show an *online* classification strategy in which the new SVD can be expressed as a product of old subspaces. These are small-matrix operations, and therefore rapid.

**Figure 6:**
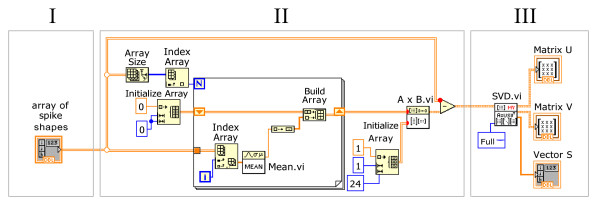
**LabVIEW programme code for centering the *X *matrix across the 2**^**nd**^**mode and SVD procedure.*I*** – *X* matrix containing spike shapes of the data set; ***II*** – centreing the matrix across the 2^nd^ mode; ***III*** – SVD procedure and resulting matrices.

Despite the good quality the standard SVD algorithm (Eq. 3) is computationally very expensive. For an *n × 24* user-item matrix, the SVD decomposition requires a run-time of *O*(*n*)^3^[14]. However, in our application it runs only once for each recording session during the period of test acquisition in unsupervised clustering analysis, while for the *online* supervised classification we use much more efficient algorithm of spike waveforms projection into the lower-dimensional space using the PSVD, as follows:

(4) $$U_{k}=XV_{k}S_{k}^{-1}\text{,}$$

where $U_{k}$, $V_{k}$_**,**_$S_{k}^{-1}$ are PSVD component matrices with *k* features, where *k* < *r* of the matrix rank. We have then reduced the problem to a lower dimensional one, the maximum number of linear independent row vectors being restricted to *k*. Such low-rank approximation of the original space filters out the small singular values that introduce “noise” subspaces and considerably improves the computational efficiency [14,27]. Once the SVD decomposition is done, the projection process involves only a dot product computation, which takes *O*(1) time, since *k* is a constant (Figure 5). The LabVIEW program code for centering the matrix and SVD is presented in Figure 6. 

### Determining the number of PCs to retain

A crucial problem in multivariate data analysis is the number of components to retain when applying PCA. This should be determined considering the tradeoff between dimensionality and the loss of data information [30]. Both underextraction and overextraction may have consequences that adversely impact the efficiency and meaning of PCA [31], resulting in classification errors. Our software solves this problem by automatically using the Scree Test Optimal Coordinates (*n*_*oc*_) method, as numerical approach to the Cattell’s scree test introduced in Ref [32]. For *online* classification we maintain the parameter obtained during test acquisition phase until our measures of goodness of clustering detect that a new training phase is needed because the data have changed their properties too much (see *Measures of cluster quality* section).

### FCM clustering and classification

**Figure 7:**
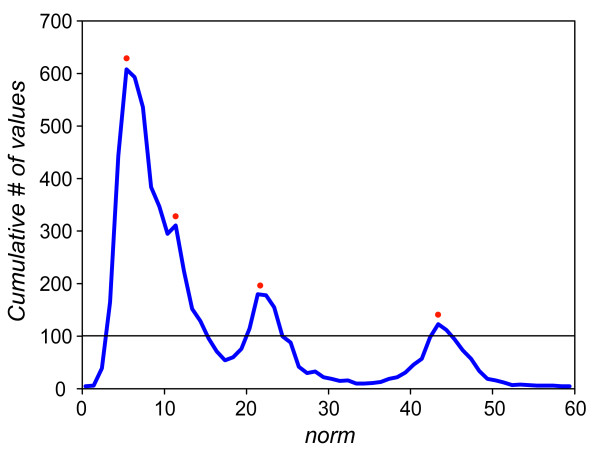
**Distribution of**$\ell_{1}$**-norm values of input singular vectors.**

Though SVD is a powerful tool for characterizing spike waveforms, it does not help to identify the neurons. It is merely a clustering technique wherein the dataset is divided into distinct clusters, which are ultimately interpreted as different single units. We have used the FCM approach based on the classical ISODATA method, using the selected above features/PCs as input variables for clustering. FCM is one of the best known and the most widely used fuzzy clustering algorithms [33]. However, due to the unsupervised nature it requires that the desired number of clusters is specified in advance. If this choice does not correspond to the actual number of clusters, the results of FCM deteriorate. In FSPS software we implemented an algorithm determining the number of clusters automatically and without supervision. To do so, we used histogram-based methods of dataset segmentation which are widely used in real-time pattern recognition systems [34]. The basic idea of algorithm we implemented in our FSPS software rests on the assumption that local densities and the number of peaks on histogram showing the distribution of $\ell_{1}$-norm values for every left singular vector in the $U_{k}$ corresponds to particular clusters (Figure 7). $\ell_{1}$-norm is considered to be generalized length (or magnitude) of the vector and calculated using the following equation:

(5) $$||X||=\left|x_{0}\right|+\left|x_{1}\right|+\ldots +\left|x_{k-1}\right|\text{,}$$

where *X* is input vector and ∥*X*∥ is a $\ell_{1}$-norm. 

Then, to construct the data-histogram we used the optimal *bin* size width *W* for the most efficient unbiased estimation of the probability density function [35], where:

(6) $$W=3.49\sigma N^{-1/3}\text{,}$$

where *σ* is the standard deviation of the distribution and *N* is the number of available samples, which corresponds to the total number of spikes or singular vectors in $U_{k}$ matrix and their $\ell_{1}$-norm values in our specific case. The histogram was than thresholded to eliminate the noise content (low-amplitude peaks in the histogram which do not correspond to any distinct cluster) by using the value of first lower valley after the mode of $\ell_{1}$-norm distribution. Thus, the number of peaks on histogram above the selected threshold was used as indication of the number of objects to be used for FCM clustering technique. However, in the interactive mode, FSPS leaves the possibility to choose the number of clusters manually, according to visual examination of the clustering results and expert judgment.

To perform FCM clustering (whose details are described in Additional file 2), one has to specify also an exponent *m* (*m* > 1.0), which determines the degree of fuzziness of the resulting clustering process. As *m*→1 the fuzziness of the clustering result tends to the results derived with the well known ISODATA method [36]. As *m*→∞ the membership values of all the objects to each cluster tend to the reciprocal of the number of classes 1/*c*. The analysis of our data obtained during recordings, the distribution of PCs and measurements of cluster quality showed that, with *m* = 1.1 the FCM algorithm is performing clustering correctly on both real and simulated data (see *Results*). Besides, this value was consistently found to lead to good results with the well- and poor- separated classes and we were able to classify the cells with acceptable accuracy. Thus, we set this default value of *m* in our program, although it can be changed by the user if needed.

### Measures of cluster quality

Since clustering algorithms define clusters that are not known a priori, it is fundamental to define a performance criterion to quantify the goodness of the resulting partition. In our FSPS software we have implemented the standard and popular figures of merit associated with FCM introduced by J.C.Bezdek (1981), such as the Partition Coefficient (*pc*), the Partition Entropy (*pe*) and Proportion Exponent (*pex*) that make use only of membership values and have the advantage of being easy to compute (see Additional file 2). However, these measures are often subject to numerical instability during the quantification of overlapping clusters of unequal size [37]. Therefore, we also included in our software one recently introduced objective validity index *L*_*ratio*_[20]. This parameter allows to obtain stable cluster evaluation from a particular recording site, and to take into account the clusters with a larger number of spikes. The evaluation of *L*_*ratio*_ changes during single unit recordings was also used as measure of classification performance to monitor the stability of data acquisition. The threshold *L*_*ratio*_ value is set to 5 as default, and can be modified by the user (we recommend a choice in the range 3–6). Once *L*_*ratio*_ becomes bigger than its threshold value, the program alerts the user that it is advised to recompute full SVD and to update FCM prototype because classification is deteriorating.

Finally, for each isolated unit the FSPS software allows to compute a number of standard quantities and statistics of interest to the neurophysiologist, such as Peri-Stimulus Time Histograms (PSTHs), raster plots and inter-spike interval (ISI) and some clinically important indices that measured tonic and phasic activity, including burst index (BI) and pause index (PI) [38].

## Results

### Performance on simulated data

In order to validate our spike sorting approach and to compare it with other known algorithms, we tested it on simulated datasets described by Quiroga et. al. (2004) and compared to their already published results obtained by superparamagnetic clustering (SPC) and K-mean clustering techniques applied to different spike features (wavelets, PCA, using the first three PCs, and the whole spike shape) [10]. The dataset contains two types of spike shapes: noisy “non-overlapping” spike shapes, which were generated by taking the target waveform and adding noise, and “overlapping spikes” which were generated overlapping spikes with a latency shorter than 0.7 ms and then adding noise. Performance was quantified in terms of number of classification errors.

**Figure 8:**
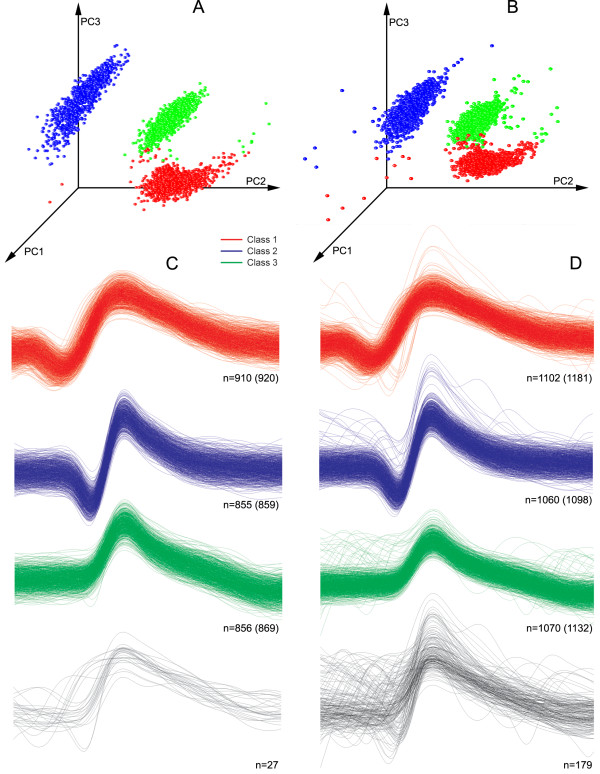
**Result of classification of the simulated dataset from Example 2, noise level 0.15.*A*** - The projection of the first three PCs of the dataset not containing overlapping spikes; ***B*** - the same, but with overlapped spikes. Three dense clusters are shown in both cases. ***C*** and ***D*** - original spike shapes of detected clusters in the datasets without and with overlapping spikes, respectively. Different spike events are shown in different colours, according to the outcome of the clustering algorithm. The number of correctly identified spikes is indicated. Values in brackets indicate the total number of spikes in each class. The spike shape shown in the lower part of the figure has been incorrectly classified and therefore marked in black.

Table 1 shows the number of classification errors of the FSPS algorithm and the other tested algorithms when detecting and sorting noisy non-overlapping spikes. FSPS gave the lowest number of false matching spikes in most simulated datasets and did not exceed 2% up to noise level 0.2 in all examples with exception of Example 4, where 7.2% of mismatches were detected. However, even in this case the outcome of FSPS technique was still in 4,5-8,8 times better compared to other methods. The advantage of FSPS becomes apparent when spike shapes are more similar (Table 1, Examples 3 and 4, considered more difficult for clustering), while our results were competitive with those obtained using K-means or SPS clustering on wavelets in Examples 1 and 2, where spike shapes of three simulated neurons were markedly different. A nice feature of the performance of our FSPS algorithm was that it degraded gracefully with increasing noise, in part due to the better outlier identification of fuzzy clustering, and the performance was reasonably good also in the case of overlapping spikes (Table 2). The reason for this improved performance is probably due to better pre-processing strategy that we employed rather than the different clustering procedure. In particular, we verified the alignment procedure and the implementation of PSVD on the clustering performance. Figure 8 illustrates this point by depicting results of classification after clustering of simulated Example 2 with noise level 0.15, a dataset that was particularly difficult to cluster with the traditional 3 PCs clustering method [10]. With our procedure, the distribution of first three PCs at the fragments A and B for the datasets without (Figure 8A) and with (Figure 8B) overlapping spikes demonstrates three clean, compact and well distinguished clusters. The presence of overlapping spikes in the dataset B (763 out of 3411, that is 22,4%) creates less distant and more shaped clusters having complex outliers. 

**Table 1:** Number of classification errors and noise levels, obtained using FSPS, SPC and K-means, in all simulated examples

**№**	**Example no.**	**Noise level**	**Number of noisy spikes**	**Classification errors**					
				**SPC**			**K-means**		**FSPS**
				**Spike Shape**	**PCA**	**Wavelets**	**PCA**	**Wavelets**	**PSVD**
		**1**	**2**	**3**	**4**	**5**	**6**	**7**	**8**
1.	1	[0.05]	2729	0	1	1	0	0	0
2.		[0.10]	2753	0	17	5	0	0	0
3.		[0.15]	2693	0	19	5	0	0	1
4.		[0.20]	2678	24	130	12	17	17	47
5.		[0.25]	2586	266	911	64	68	69	157
6.		[0.30]	2629	838	1913	276	220	177	221
7.		[0.35]	2702	1424	1926	483	515	308	354
8.		[0.40]	2645	1738	1738	741	733	930	462
9.	2	[0.05]	2619	2	4	3	0	0	0
10.		[0.10]	2694	59	704	10	53	2	2
11.		[0.15]	*2648*	1054	1732	45	336	31	27
12.		[0.20]	2715	2253	1791	306	740	154	48
13.	3	[0.05]	2616	3	7	0	1	0	0
14.		[0.10]	2638	794	1781	41	184	850	0
15.		[0.15]	2660	2131	1748	81	848	859	17
16.		[0.20]	2624	2449	1711	651	1170	874	22
17.	4	[0.05]	2535	24	1310	1	212	686	0
18		[0.10]	2742	970	946	8	579	271	7
19.		[0.15]	2631	1709	1716	443	746	546	51
20.		[0.20]	2716	1732	1732	1462	1004	872	195
*Average*			*2663*	*874*	*1092*	*232*	*371*	*332*	*81*

Noise level is represented in terms of its standard deviation relative to the peak amplitude of the spikes. All spike classes had a peak value of 1. The absolute number of false matching spikes is shown in the column 8 as the outcome of our algorithm corresponding to the datasets containing noisy spikes (column 2).

**Table 2:** Number of classification errors for all simulated examples and overlapping spike shapes

**№**	**Example no.**	**Noise level**	**Number of overlapping spikes**	**False matches**	
				**N°.**	**%**
		**1**	**2**	**3**	**4**
1.	1	[0.05]	785	161	20.5
2.		[0.10]	769	146	19.0
3.		[0.15]	784	185	23.6
4.		[0.20]	796	165	20.7
5.		[0.25]	712	208	29.2
6.		[0.30]	846	250	29.6
7.		[0.35]	832	270	32.5
8.		[0.40]	741	270	36.4
9.	2	[0.05]	791	152	19.2
10.		[0.10]	826	167	20.2
11.		[0.15]	*763*	152	19.9
12.		[0.20]	811	301	37.1
13.	3	[0.05]	767	88	11.5
14.		[0.10]	810	131	16.2
15.		[0.15]	812	152	18.7
16.		[0.20]	790	287	36.3
17.	4	[0.05]	829	39	4.7
18		[0.10]	720	114	15.8
19.		[0.15]	809	209	25.8
20.		[0.20]	777	282	36.3
*Average*			*789*	*186*	*23.7*

Noise level is represented in terms of its standard deviation relative to the peak amplitude of the spikes. All spike classes had a peak value of 1. The absolute number of false matching spikes is shown in column 3 as the outcome of our algorithm corresponding to the datasets containing overlapped spikes (column 2).

### Description of real multi-unit data

We then tested our spike sorting algorithm and its quality of separation using real polyspikes recorded from premotor area F5 of a Macaque monkey. To demonstrate the functionality of our method in a realistic situation we present spike sorting results of two different datasets, obtained from the same recording site, but in different experimental conditions, thus introducing the complexity of non-stationarity in the data. First dataset, referred to as A, was acquired when monkey performed goal-directed grasping movements in full vision, while other recordings (referred to as dataset B) were done 30 min after A during the same grasping in the dark. Each dataset contains raw signals of twelve 3 s trials of each of the two conditions with a 10-12 s intertrial interval. The total duration of acquisition of one dataset was therefore 2–3 min. The structure of the two datasets and the result of spike identification are shown in Additional file 2: Table S1 (dataset A) and Additional file 2: Table S2 (dataset B). In the waveform extraction phase, 475 and 295 false positive peaks exceeding the threshold value were automatically removed from datasets A and B, respectively. Thus, 6191 spikes out of 6666 identified in the dataset A and 6379 out of 6674 in the dataset B were processed. Dataset A was used at first as test acquisition for the unsupervised spike sorting, creation of SVD model (*U*_*k*_, *S*_*k*_, and *V*_*k*_) and to obtain the parameters that were necessary to further supervised spikes classification of the dataset B.

### SVD and pre-processing results on real multiunit recordings

**Figure 9:**
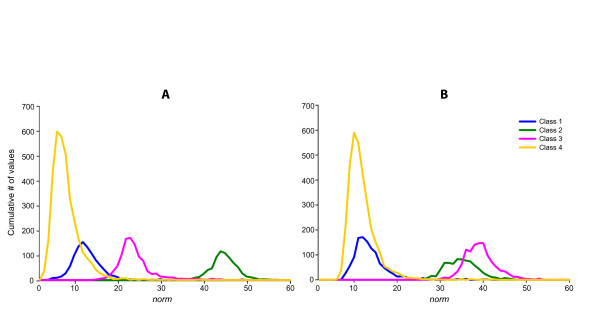
**Distribution of**$\ell_{1}$**-norm values of input singular vectors for every Class.** This figure shows the frequency distribution of $\ell_{1}$*-norm* values *of singular vectors* for each determined class in datasets ***A*** and ***B***, respectively. Different classes are shown in different colours, according to the outcome of the clustering algorithm.

**Figure 10:**
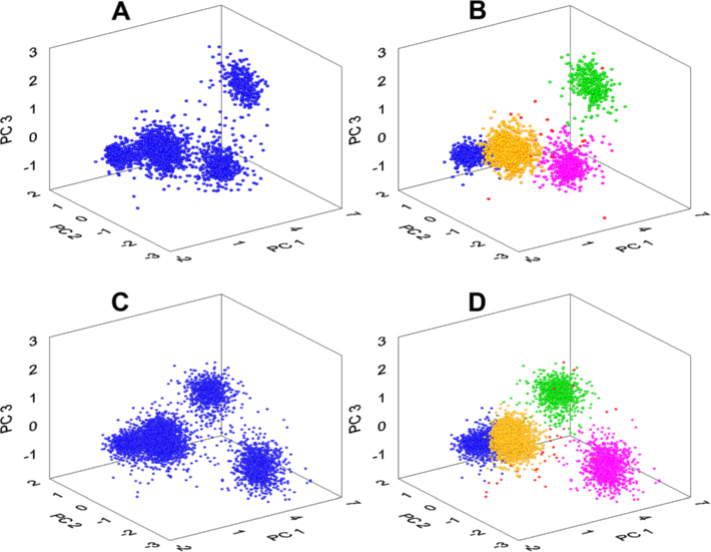
**Rotable 3D scatter-plots of first three PC scores.** The Figure shows an example automatic clustering of dataset A (fragments *A* and *B* are conditions before and after clustering analysis) and automatic classification of dataset B (fragments *C* and *D* are conditions before and after clustering). Each dot represents a spike situated in the 3D PC feature space. Four dense clusters are clearly visible and classified. Noise-near waveforms and their PCs located far away from cluster centres, as evaluated by Mahalanobis distance, are marked as red dots.

**Figure 11:**
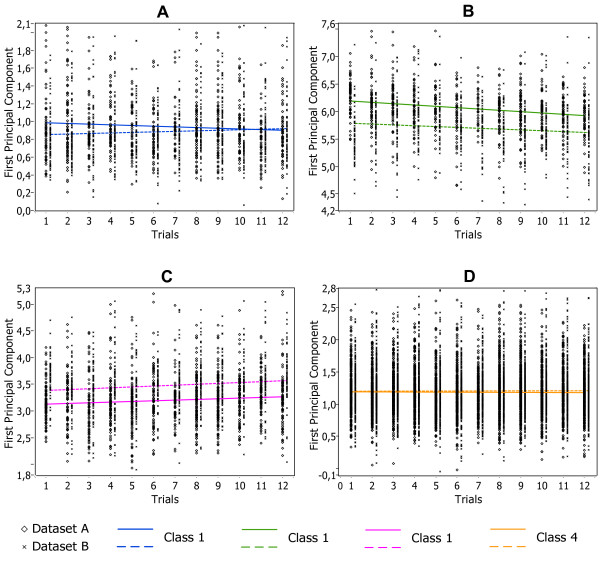
**Time course of clusters over recording trials.** This Figure shows how the waveform moves along the axis of maximum variance. The trend represents the spike waveform changing shape due to electrode drift. Clusters 1–4 are shown in Fragments A-D, respectively.

The implementation of full SVD (Eq.2) on the dataset A gave origin to matrices *U*^(6191×24)^, *S*^(24×24)^ and *V*^T (6191×24)^. The elaboration of singular values in diagonal *S* matrix by algorithm determining the optimal number of features detected *n*_*oc*_ *= 4 PCs* accounting 67,3% of total variance*.* Then, the software calculates $\ell_{1}$-norm values for every left singular vector (contains PCs) composing the matrix $U^{\left(6191\times 4\right)}$. The distribution of these $\ell_{1}$-norm values showed four detectable peaks on the line-graph histogram (see Figure 9A), thus four clusters available at the PCs feature space (Figure 10A,B). The matrix *V*^T^ and other parameters for the input of FCM clustering algorithm found on dataset A were kept in memory and then used to classify the spikes of dataset B. The *L*_*ratio*_ measure of quality of clustering remained below the threshold value of 5 (Table 3) and so the software algorithm did not detect the need to repeat the testing phase of the clustering algorithm until the end of experiment B. The 3D scatter plot of first three PCs of dataset B shows same four clusters (Figure 10C). While two dense and partially overlapping clusters, located in the leftmost part of each plot, seem to be almost identical, the other two become more spread and shifted in 3D space in the dataset B with respect to dataset A, as it follows also from the Figure 11 representing the time course of clusters. We projected these clusters onto PCs axis of maximum variance and plotted this projection for each trial that were recorded sequentially. Figure 11B shows that a negative trend occurs in the projection, while Figure 11C shows the positive one. The solid and dash lines represents the centroid of the cluster as it changes over time for dataset A and B, respectively. The points in the figure represent a single spike waveform changing shape slowly and continuously. The changes in the internal structure of the dataset B become more evident considering the distribution of $\ell_{1}$-norm values of singular vectors depicted in the line-histogram at the Figure 9B as Classes 1 and 4 become completely overlapped. Despite this fact all clusters are still well recognized and associated correctly by our approach thanks to cluster information previously learned in dataset A (Figure 10D).

**Table 3:** Performance of FSPS™ clustering/classification of datasets A and B with their respective quality measures

**Parameter**		**Value**	
		**Dataset A**	**Dataset B**
		**/clustering/**	**/classification/**
Number of spikes processed		6191	6379
Average processing time (at our system), ms		684 ± 21	44 ± 1
Number of Steps		34	1
Training Error		0.00002	0.00002
Test Error		0.00000	0.00000
Partition Coefficient (*PC*)		0.99208	0.98411
Partition Entropy (*PE*)		0.01313	0.02667
*L*_*ratio*_	Class 1 (blue)	2.478	2.504
	Class 2 (green)	4.180	4.243
	Class 3 (pink)	2.626	2.677
	Class 4 (yellow)	0.493	0.500

**Figure 12:**
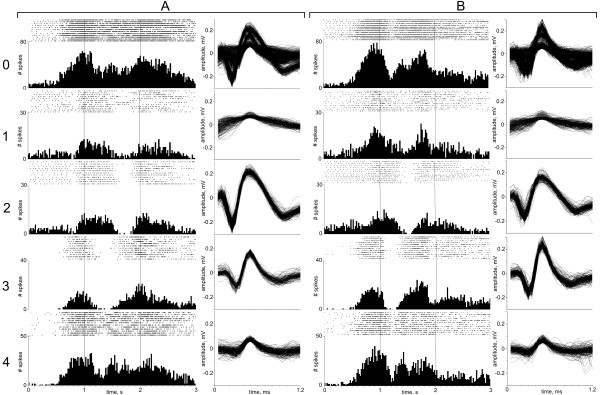
**Validation of waveforms in determined Classes with corresponding histograms and rasters.** This Figure shows how the outcome of classification of datasets A and B, respectively, can be backwards applied to the raw signals to build rasters and histograms describing individual neurons’ response during the experimental task. Notes: *0* – all spikes, Unclassified; *1* – Class 1, *2* – Class 2; *3* – Class 3; *4* – Class 4.

To prove that the outcome of PCs clustering analysis/classification of the datasets is successful as well as obtained classes are assigned same single units, the results were backwards applied to the raw signals to build rasters and histograms describing individual neuron response (Figure 12). The firing properties were consistent across the two datasets suggesting that the units classified in dataset B are the same as those discovered in dataset A, and so the FSPS software can accurately track neurons despite non-stationarities in the data. Besides, the robustness of our method is demonstrated by comparing clustering results of two types of high-amplitude discharges, isolated as Class 2 and Class 3 in four PCs features space, and having specific reciprocal electrophysiological behaviour (see PSTHs and rasters in Figure 12).

**Figure 13:**
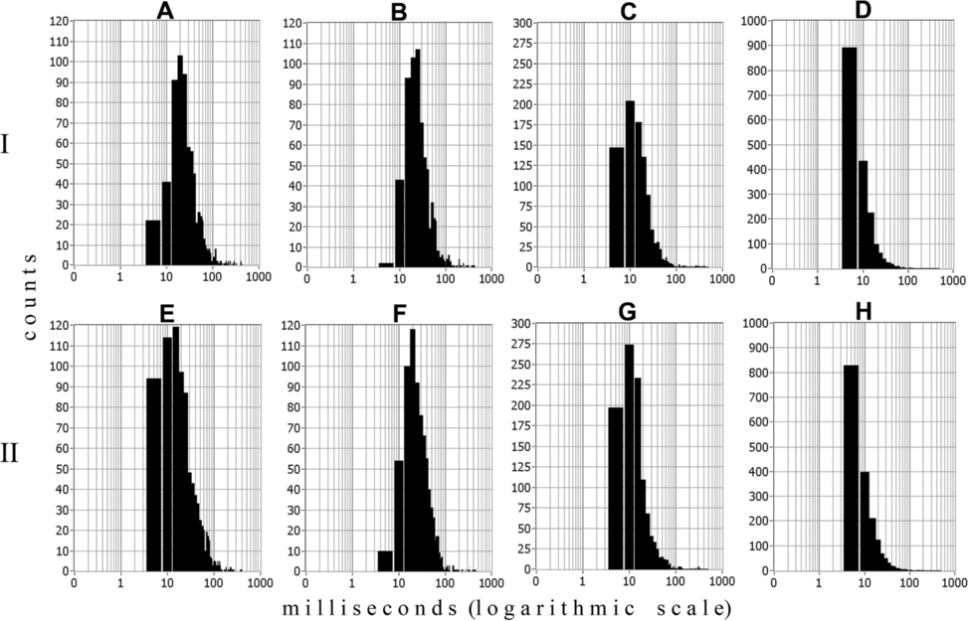
**Effects of sorting in inter-spike interval (ISI) distributions.** Fragments *A–D* show the true ISI distribution of the timestamps for classes 1–4 of dataset A, respectively, determined by PCs clustering analysis Fragments *E–H* show the ISI distributions for the timestamps attributed to classes 1–4 of dataset B, determined by the PCs classification procedure. *I* – dataset A, *II* – dataset B. Given examples shows no events in the refractory period.

Despite the fact that the amplitude of their discharge has been mismatched in dataset B, thus making unfeasible amplitude-based sorting, they are still well separated by our technique because it takes into account the whole profile of spike shape. The analysis of spike times and ISI histograms of isolated neurons in both datasets shows no multi-unit contamination (Figure 13).

### Exploring the limits of classification and its reliability for the online spike sorting

It is known that FCM algorithms have a tendency to find clusters of comparable size because they use a sum of squared errors objective function and approximately equal cluster populations result in smaller values of this objective function [17,39]. This might become a serious problem in *online* applications, where the algorithm may be applied to relatively small stretches of data and so random fluctuations of spike rates may make the relative size of clusters at a given time very disproportionate.

**Figure 14:**
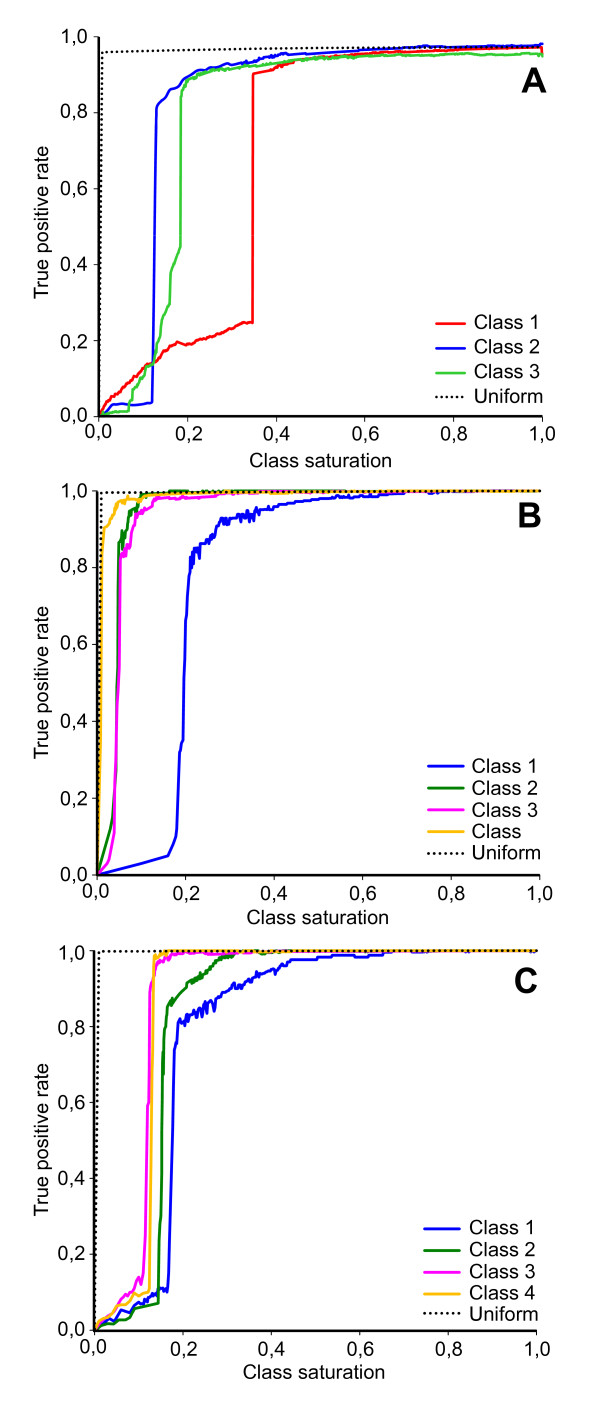
**Limits of classification capability for the simulated and real datasets.*A*** - ROC graph showing the performance of the FCM-classifier during balanced (dotted line) and unbalanced modification (solid lines) of classes in the simulated dataset; ***B*** – the same for the real dataset A; ***C*** - the same for the real dataset B.

To investigate this issue, in this section we evaluated the robustness of datasets classification to disparity in cluster size, by progressively eliminating spikes in a cluster and computing the performance of the clustering algorithm as function of the class saturation, i.e. of the fraction of spikes left in the cluster. Results are shown in Figure 14, showing the simulated data and the real datasets A and B. Color lines show the true positive rate (i.e. the percentage of spikes retaining true cluster membership) when reducing the size of a particular cluster while the size of other clusters remains unchanged. The accuracy of classification of the simulated Example 2 with noise level 0.15 is shown in Figure 14A. Unbalanced decrease of clusters up to 40% of their original size shows still high classification accuracy (right-hand side of the graph). Further cluster decrease shows minor deterioration of classification accuracy due to drifting of smaller clusters toward lager adjacent ones. An abrupt and pronounced deterioration in the partitioning of the data was found only when clusters 1, 2 or 3 remain less than 34,7%, 18,5% or 14,1%, of their original size, respectively.

Figure 14B shows the results obtained with real dataset A. The classifier is still performing well and true positive rate is higher 80% if it remains at least 20,1%, 4,9%, 1,1% and 5,1% spikes, respectively to Class 1, Class 2 Class 3 and Class 4. Figure 14C shows the outcome of the classification real dataset B. The true positive rate for each class remains still at higher than 80%, if classes 1, 2, 3 or 4 contain at least 18,9%, 16,1%, 13,3% and 12,5% spikes, respectively.

An additional exploratory test in which all clusters were modified in a balanced and “Uniform” manner showed excellent performance in simulated, real A and real B datasets (black dotted line in Figure 14A-C). In all these cases the classification accuracy was completely independent of the density of the clusters and the true positive rate was at its maximal initial value until the each cluster contained at least one spike.

## Discussion

The present work is devoted at addressing the challenges involved in balancing the different needs for accuracy, speed and automation in spike sorting.

The first point of discussion regards the selection of the optimal spike-shape features to be used for sorting [1,40-42]. Here we chose the multivariate techniques for selection of spike features. The multivariate approach has proven successful in many industrial *online* applications [15,43]. In this work, it is shown that neurophysiological research does not constitute an exception. We improved the performance of PC based classification by using a careful alignment of spike shapes and PSVD to reduce noise and computational time and to select the optimal number of components to be used, thereby choosing a reduced variable sets as inputs for the clustering algorithms. In agreement with other studies [8,44-46], we have indeed found that when careful alignment and PCA were implemented, the results of classification of simulated datasets showed high tolerance to the noise, with better performance in comparison with similar methods.

Another goal of this paper was to demonstrate the applicability and efficacy of our technique for *online* isolation of single neurons. Indeed, a straight PCA approach is not sufficient to describe the changing pattern of neural activity adequately [11,13]. There are mainly two reasons for this. First, the neuronal activity displays a non-stationary behaviour [47]. Second, PCA is not an optimal method for feature extraction when the features are used in a supervised classifier [27]. Finally, PCA is a computationally intensive preprocessing technique, making hard its use in real-time processing. Despite these conceptual difficulties our way of implementation of the mentioned algorithms into LabVIEW environment allowed us to run *online* classification with small and acceptable delay as far as our experimental conditions are concerned. There are several examples of applications of multivariate statistical *online* monitoring (and modeling) associated with PCA and FCM overcoming problems associated with non-stationarity [48-50].

A main focus of this article was the accuracy assessment of the FCM, which have been incorporated into FSPS software to produce both crisp and fuzzy classifications. The main advantage of FCM implementation is that fuzzy classification gives faster detection and smoother control than crisp classification. The importance of this becomes more evident during *online* classification. The tests reported in this article suggest that the FCM clustering copes well with the problems generated by the non-stationarity of the real data. It is interesting to note that the FCM procedure is based on an iterative clustering algorithm and can thus be regarded as an essentially unsupervised classifier. However, we also implemented a partially supervised mode, benefitting from partition and membership function previously obtained from training dataset. Consistently with this, the results of our tests performed on datasets prove that the implementation of FCM overcomes the problem of sensitivity for unequal cluster sizes, which is crucial for correct *online* classification. Thus, together with PCs extracted by PSVD from accurately preprocessed spike waveforms, FCM becomes a versatile noise tolerant technique for the sorting of neuronal action potentials having even small variation in their discharge.

Any kind of clustering or classification needs an objective measure of its quality. Although we implemented in the FSPS software most conventional indices associated with FCM, including *partition coefficient*, *partition entropy* and *proportion exponent*, our tests showed that the index *L*_*ratio*_ was superior to classic FCM indexes and so was implemented as the default quality measure in our software. In terms of real recordings we found *L*_*ratio*_ useful not only to determine whether the quality of a cluster is within acceptable limits, but also to control the stability of recordings, to predict the future behavior of the neuron and check whether the SVD model is going out of control.

An important feature of the FSPS software is that it is implemented entirely within LabVIEW. The latter constitutes one of the most frequently used programming languages for the data acquisition, analysis, control and visualization. LabVIEW is often faster than many other high-level programming languages used in neuroscience, such as MATLAB [22], and it is far better equipped for the development of experimental and clinically-oriented spike sorting applications [51]. The upshot is that the entire spike time acquisition process can be run within a single environment, which has the all-important added benefit of simplifying experimental procedures.

In recent years there have been successful attempts at creating a cross-platform GUI for data visualization, navigation and spike sorting features within another software environment using the Python framework [52,53]. Python applications, like “SpikeSort”, “Spikepy”, “spyke” and “OpenElectrophy”, provide adequate tools for the exploration of data and offline spike sorting, while “NeurOnline” provides the means for online spike sorting. However, the LabVIEW code we used is far more convenient because it rarely calls the Operating System (OS) directly, so it can be used with different OSs without the need for major modifications. Moreover, LabVIEW supports thousands of hardware devices and, in addition to the popular desktop OSs (Windows, Mac, and Linux), it can target several embedded real-time controllers, ARM microprocessors, and field-programmable gate arrays (FPGAs), allowing the deployment of our FSPS code with the most appropriate hardware platform without the need to learn new toolchains.

All mentioned properties contributed to the creation of a fast, powerful, user-friendly and stand-alone multi-platform software, designed for clustering/classification of neural data. Moreover, our *online* approach will help physiologist to overcome new challenges in experimental electrophysiological research.

## Conclusions

We believe that the software developed here complements existing spike sorting toolboxes and will be a useful tool for fast *on-* and *offline* sorting of spike trains with limited supervision or fully automated. Because of these properties, our tool will be particularly useful for the analysis of large parallel recordings (where human supervision is practically impossible or inconvenient) and will therefore be important for improving our understanding of population codes [54-56] and for *online* applications such as the decoding of neural ensembles to control Brain Computer Interfaces or for clinical applications.

## Availability and requirements

Project name: Neurolab

Project home page: http://www.spikesorting.com

Operating system(s): It was tested on Windows XP, Windows Vista, Windows 7

Programming language: NI LabVIEW 2009

Other requirements: for running in the *online* mode, the requirements are as follows. Hardware: Digital acquisition board from National Instruments (PCI or USB). Additional software: 1. LabVIEW Run-Time Engine 2009 for Windows 2000/7/7 x64/Vista/Vista x64/XP - (32-bit Standard RTE) - available free at: http://www.ni.com. 2. NI-DAQmx Run-Time Engine 9.3 or higher - (Core) for Windows 7 64 bit/7 x86/Server 2003 R2 (32-bit)/XP x86/Vista x64/Vista x86/Server 2008 R2 (64-bit) - available free at: http://www.ni.com;

License: FSPS software is distributed under Creative Commons Public License (CCPL BY-NC-ND) and can be used for non-commercial academic applications providing they properly reference this work in any publication that uses results generated by FSPS software.

Any restrictions to use by non-academics: Commercial License needed.

## Abbreviations

BI: Burst index; DAQ: Digital Acquisition; FCM: Fuzzy C-mean; FPGA(s): Field-programmable Gate Array(s); FSPS: Fuzzy SPike Sorting; GUI: Graphical User Interface; ISI: Inter-spike Interval; OS(s): Operating System(s); PC(s): Principal Component(s); PCA: Principal Component Analysis; PI: pause index; PSTH(s): Peri-Stimulus Time Histogram(s); PSVD: Partial Single Value Decomposition; SPC: Superparamagnetic Clustering; SVD: Single Value Decomposition; VI(s): Virtual Instrument(s).

## Competing interests

The authors declare that they have no competing interests.

## Authors’ contributions

AO - conceived, refined and implemented the algorithm, developed and designed software, performed single-unit recordings, analyzed simulated data, evaluated results, wrote the draft of the paper and created the website. CB - contributed to the theoretical developments of spike sorting algorithm. FM - tested the software on simulated data. LF - conceived the project, refined the software requirements and co-wrote the paper. All authors read, commented upon, and approved the final manuscript.

## Supplementary Material

Additional file 1 This file contains the FSPS Manual and Installation Notes. Click here for file

Additional file 2 This file contains supplemental text, figure and tables, which corroborate the findings presented in the main text. Click here for file
