# Supplementary material for: Automatic online spike sorting with singular value decomposition and fuzzy C-mean clustering
Source: BMC Neurosci. 2012 Aug 8;13:96. doi: 10.1186/1471-2202-13-96 (PMC3473300; doi:10.1186/1471-2202-13-96)
Supplement: Additional file 1 — This file contains the FSPS Manual and Installation Notes. [file 1471-2202-13-96-S1.pdf]

Supplemental Information for

Automatic *Online* Spike Sorting with Singular Value Decomposition and Fuzzy C-mean Clustering

Andriy Oliynyk, Claudio Bonifazzi, Fernando Montani and Luciano Fadiga

S1. Resolving the false-positive issue

The strategy of removing false-positive spikes is based on the assumption that most of them derive from various phases of one action potential (as clearly shown in Figure below), thereby representing one neuronal discharge. If more than one spike is detected within the time-window (0.8 ms in our acquisition), the additional low-amplitude detections are counted as false positives and cancelled at the extraction/interpolation stage.

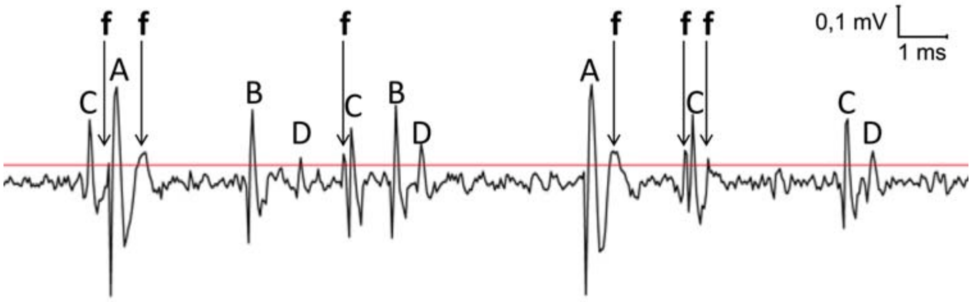

Notes: *A*-*D* – spike events arising from different single units, *f* – false positive events.

On the other hand, true synchrony is also possible. The probability is:

$$\%\text{missed spikes} = 100rd / 1000 \qquad \text{(Lewicki 1998)}$$

where r is the firing rate in Hertz and d is the duration of the negative phase of action potential in milliseconds. Hence, if the background neuron is firing at 5 Hz (an

average for cortical neurons) and the duration of the negative phase is approximately 0.5 ms, then approximately 0.25% of the spikes will be missed. This level is considered negligible.

S2. FCM clustering algorithm

A.1. Initialize the membership values μ_{ik} of the k objects x_k to each of the i clusters for k=1,etc., K randomly, so that:

$$\sum_{i=1}^{c} \mu_{ik} = 1, \quad \forall k = 1,\ldots,K \text{ and } \mu_{ik} \in [0,1] \quad \begin{matrix} \forall i = 1,\ldots,c \\ \forall k = 1,\ldots,K \end{matrix} \qquad \text{(A.1)}$$

A.2. Calculate the cluster centres v_i using the following membership values μ_{ik}:

$$v_i = \frac{\sum_{k=1}^{K} (\mu_{ik})^m \cdot x_k}{\sum_{k=1}^{K} (\mu_{ik})^m}, \quad \forall i = 1,\ldots,c \qquad \text{(A.2)}$$

A.3. Calculate the new membership values μ_{ik}^{new} using these cluster centres v_i:

$$\mu_{ik}^{new} = \frac{1}{\sum_{j=1}^{c} \left(\frac{\|v_i - x_k\|}{\|v_j - x_k\|} \right)^{\frac{2}{m-1}}}, \quad \begin{matrix} \forall i = 1,\ldots,c \\ \forall k = 1,\ldots,K \end{matrix} \qquad \text{(A.3)}$$

A.4. If $\left\| \mu^{new} - \mu \right\| > \varepsilon$, let $\mu^{new} - \mu$ and go to step A.2. (A.4)

The Euclidean distance was used in calculation of the vector distances in step A.3. The process ends when the distance between two successive membership matrices μ falls below a stipulated convergence threshold ε.

In addition to providing the position of the cluster centres v_i, with the aid of step A.3 the FCM algorithm also provides the membership values of the individual objects to the different clusters. This permits classification of new objects and their membership

values to the different classes for the given cluster centres. In order to be able to apply the detected cluster centres as classifiers, these need to be given recognizable names. The linking of the class names to the cluster centres is achieved using a scoring matrix. Given c clusters and a set of PCs x_k, each belonging to a cluster $class(k) \in \{1,...,c\}$, a scoring matrix P is given of dimensions $c \times c$ whose elements are initialized as 0.0. The cluster names are then automatically assigned to class centres using the following algorithm:

A.5. Calculate the membership function μ_{ik} of the object x_k of *class(k)* to all the cluster centres v_i:

$$\mu_{ik} = \frac{1}{\sum_{j=1}^{c}\left(\frac{\|v_i - x_k\|}{\|v_j - x_k\|}\right)^{\frac{2}{m-1}}}, \quad \forall i = 1,...,c \qquad \text{(A.5)}$$

A.6. Let $P_{class(k),i} = P_{class(k),I} + \mu_{ik}, \quad \forall i = 1,...,c$ (A.6)

A.7. Go to step A.5 until all examples x_k have been processed by step A.5 and A.6.

A.8. Determine $l_i = \max_{k=1,...,c}\{P_{k,i}\}$ for $i=1,\dots,c$.

A.9. Assign the label l_i to the cluster center v_i.

We use a slightly modified version of the algorithm above, which offers distinct advantages as regards the computing time and memory required, despite being equivalent in procedure to that described.

In place of the membership matrix, the algorithm initializes the matrix of the cluster centres. For this reason, steps A.2 and A.3 of the above algorithm are swapped around. The process ends by comparing two successive cluster centre matrices, the matrix *norm* being the sum of the vector components.

In addition to providing the position of the cluster centres, with the aid of step A.3 the FCM algorithm also provides the membership values of the individual objects to the

different clusters. This permits classification of new objects and their membership values to the different classes for the given cluster centres. This is the core of our *online* FCM-classifier, in which all new PCs are calculated with respect to known (obtained during test acquisition) class centres.

S3. Cluster validity measures

The partition coefficient is a measure of the crispness of clustering and/or of the ability of the individual clusters to be distinguished from one another. Higher values indicate crisper cluster results.

Partition Coefficient (*pc*): $$pc = \sum_{k=1}^{K}\sum_{i=1}^{c}\frac{(\mu_{ik})^2}{K}$$ (B.1)

The Partition Entropy is an indicator of the disorder in the object set with regard to the resulting classifier. Small values indicate more order.

Partition Entropy (*pe*): $$pe = -\frac{1}{K}\sum_{k=1}^{K}\sum_{i=1}^{c}\mu_{ik}\cdot\ln(\mu_{ik})$$ (B.2)

The Proportion Exponent value is also a measure of the crispness of clustering. However, only the maximum memberships of an object to a cluster are taken into account here. The proportion exponent is not defined if at least one object is crisply assigned to a cluster (membership of 1.0).

Proportion Expone*nt (pex):* $$pex = -\ln\prod_{k=1}^{K}\sum_{i=1}^{m_k}(-1)^{i+1}\binom{c}{i}(1-i\cdot\mu_k)^{c-1}$$ (B.3)

Supplemental Tables

Supplemental Table 1 - The structure of data set A by BINs through trials.

		BINs												
		**1**	**2**	**3**	**4**	**5**	**6**	**7**	**8**	**9**	**10**	**11**	**12**	**Total**
**TRIALS**	**1**	13/1	16/1	45/5	71/3	73/6	43/6	55/1	71/13	80/2	56/3	36/2	36/2	**595/45**
	**2**	13/1	16/1	34/1	85/3	68/4	42/2	50/0	56/5	73/5	62/5	53/8	19/1	**571/36**
	**3**	6/1	7/0	21/1	81/6	73/3	32/1	40/1	58/5	82/7	59/4	30/2	17/0	**1166/81**
	**4**	17/1	6/0	62/4	84/4	61/7	45/3	38/1	62/3	83/8	49/2	15/1	27/1	**549/35**
	**5**	7/1	11/1	51/5	70/3	78/5	63/7	52/4	43/1	58/5	82/9	33/1	24/0	**572/42**
	**6**	3/0	3/0	17/2	64/2	83/10	53/3	54/8	63/6	42/0	71/2	57/5	28/3	**1121/77**
	**7**	10/2	16/2	43/1	71/5	50/4	48/3	32/1	64/3	22/1	14/0	17/0	5/0	**392/22**
	**8**	14/2	15/1	35/2	87/6	63/3	45/5	57/2	62/4	74/6	42/3	69/8	17/0	**580/42**
	**9**	13/1	15/2	41/2	77/8	82/5	52/1	63/4	43/1	93/8	87/6	73/8	29/4	**972/64**
	**10**	13/2	20/2	27/1	83/7	73/7	49/5	42/4	58/4	84/6	72/4	27/1	13/0	**561/43**
	**11**	9/1	20/4	36/3	85/7	72/10	46/3	41/3	73/9	78/5	47/4	26/1	28/0	**561/50**
	**12**	8/1	8/1	42/2	68/3	87/4	43/5	46/1	51/4	82/6	56/4	48/6	34/1	**1122/93**
	**Total**	**126/14**	**153/15**	**454/29**	**926/57**	**863/68**	**561/44**	**570/30**	**704/58**	**851/59**	**697/46**	**484/43**	**277/12**	**6666/475**

Supplemental Table 2 - The structure of data set B by BINs through trials.

		BINs												
		**1**	**2**	**3**	**4**	**5**	**6**	**7**	**8**	**9**	**10**	**11**	**12**	**Total**
**TRIALS**	**1**	10/0	13/0	48/3	99/3	77/3	50/0	64/3	83/3	19/2	17/0	31/1	48/1	**559/19**
	**2**	12/0	13/0	43/4	94/7	66/3	69/0	67/4	82/3	71/3	32/1	9/0	24/0	**582/25**
	**3**	10/0	21/0	75/4	107/7	61/3	65/1	77/9	44/3	51/0	55/2	30/0	23/1	**619/30**
	**4**	18/0	25/1	52/3	93/6	65/4	61/1	68/3	71/5	45/1	44/1	33/2	31/1	**606/28**
	**5**	9/0	11/0	48/3	81/6	85/7	50/4	53/3	73/3	61/3	41/1	25/1	47/2	**584/33**
	**6**	11/0	16/1	58/2	97/6	45/1	53/3	97/7	44/1	15/0	31/1	18/0	12/1	**497/23**
	**7**	6/0	10/0	36/2	93/5	77/7	50/1	58/3	70/7	43/1	54/3	24/1	16/1	**537/31**
	**8**	8/0	5/0	41/1	111/7	53/3	64/2	72/6	53/1	47/1	38/2	61/2	59/1	**612/26**
	**9**	9/0	7/0	46/3	92/6	51/3	51/2	51/0	64/2	40/2	42/1	9/0	17/0	**479/19**
	**10**	8/0	14/0	26/1	91/4	59/3	47/1	58/5	83/5	46/3	33/1	23/1	14/0	**502/24**
	**11**	13/0	19/0	33/2	99/6	58/2	56/1	63/3	80/6	29/1	35/0	39/2	78/4	**602/27**
	**12**	10/0	7/0	23/0	89/2	57/2	49/2	69/2	79/1	43/0	40/1	18/0	11/0	**495/10**
	**Total**	**124/0**	**161/2**	**529/28**	**1146/65**	**754/41**	**665/18**	**797/48**	**826/40**	**510/17**	**462/14**	**320/10**	**380/12**	**6674/295**
