# Supplementary material for: Automatic online spike sorting with singular value decomposition and fuzzy C-mean clustering
Source: BMC Neurosci. 2012 Aug 8;13:96. doi: 10.1186/1471-2202-13-96 (PMC3473300; doi:10.1186/1471-2202-13-96)
Supplement: Additional file 2 — This file contains supplemental text, figure and tables, which corroborate the findings presented in the main text. [file 1471-2202-13-96-S2.pdf]

Rev.	Prepared by		Date
2.3.0.10	University of Ferrara, Faculty of Medicine, Department of Biomedical Science and Advanced Therapy, Section of Human Physiology	Andriy Oliynyk	**30.06.2012**

Revision History

Rev.	Date	Description
1.0.1.00	19/06/2010	Preliminary launch
1.1.0.00	19/09/2010	Update 1
1.2.7.11	22/03/2011	Update 2
1.3.1.46	12/04/2011	Update 3
1.4.0.00	20/04/2011	Update 4
1.6.0.16	14/09/2011	Update 5
2.0.0.32	31/10/2011	Update 6
2.1.0.84	24/01/2012	Update 7
2.2.0.15	01/02/2012	Update 8 – FTDI support is included
2.3.0.10	10/06/2012	Update 9 – This version introduced a number of significant enhancements including: improved classification performance suitable now for multi-electrodes; rewriting the core-code without commercial third-part add-on; improvements in visualization during continuous recordings, cluster information added, export data into .txt file, export cluster center information, some bug fixed and more**.**
3.0.0.4	30.06.2012	The implementation of Creative Commons Public License (CCPL BY-NC-ND) for academic non-commercial use **(current version)**.

Table of Contents

Revision History 1
Table of Contents 2
General Information 3
Terms & Conditions Agreement 4
Technical Requirements 6
Installation 7
Chapter 1. Getting Started 8
With NI Digital Board 8
Without NI Digital Board 9
Chapter 2. Continuous Signal Acquisition and Off-line Spike Sorting 10
Chapter 2.1 (Control Tab 0, Continuous or Triggered Acquisition) 11
Chapter 2.2 (Control Tab 1, Basic I/O Parameters, Control Tab) 12
Chapter 2.3 (Control Tab 2, FCM Status and Performance Information) 15
Chapter 2.4 (Control Tab 3, Visualization and Statistics) 16
Chapter 3. Triggered Acquisition and Off-line Spike Sorting 23
Chapter 3.1 (Control Tab 0, Continuous or Triggered Acquisition) 25
Chapter 3.2 (Control Tab 1, Hardware Info & Settings) 25
Chapter 3.3 (Create New Set) 27
Chapter 3.4 (Get Instant Principal Components) 28
Chapter 4. On-line Spike Sorting 29
Chapter 4.1 (Input/Output Parameters) 31
Chapter 4.2 (Flow Chart) 31
Chapter 4.3 (HDD Streaming) 32
Examples and Supporting Files 33
FAQ & Troubleshooting 34
Glossary 35

General Information

A challenging issue in computational neurosciences is the development of algorithms to detect and sort the spiking activity of individual neurons from extracellular recordings. While many algorithms for spike sorting exist, the problem of accurate and fast online sorting of spikes still remains to be solved.

In answer to this problem, this novel software tool, FSPS™ (Fuzzy SPike Sorting), has been designed to optimize fast and accurate detection, off-line sorting and on-line classification of neuronal spikes with little or no human intervention.

The FSPS™ algorithm is based on a combination of the following main methods:

a) SPLINE interpolation for high-resolution alignment of extracted spike waveforms;
b) Singular Value Decomposition (SVD) and its modification Partial Singular Decomposition (PSVD) for fast and highly accurate *online* pre-processing of spike shapes;
c) Scree Test Optimal Coordinates (SOC) for optimal selection of the number of features to retain;
d) Automatic identification the number of clusters using a histogram-based method of dataset segmentation;
e) Fuzzy C-mean (FCM) for the clustering/classification of PCs in the multi-dimensional space feature;
f) Mahalanobis distance measurements;
g) Quantitative quality assessment of the resulting clusters, irrespective of their size.

The software was entirely written and compiled using LabVIEW™ 2009 (National Instruments, USA).

FSPS™ software is available at http://www.spikesorting.com

Terms & Conditions Agreement

FSPS^{TM} is a computer programme for the fast and accurate automatic isolation of neurons during electrophysiological recordings; it can be used for signal acquisition, visualization, classification and analysis of neuronal spikes. For more information regarding the current version of FSPS^{TM} please e-mail: info@spikesorting.com.

Prior to download and use, the user acknowledges that:

1. FSPS™ is distributed under Creative Commons Public License (CCPL BY-NC-ND) and can be used for academic applications providing they properly reference our work in any publication that uses results generated by FSPS™ software. Under this CCPL you are free:
to Share - to copy, distribute and transmit the work

- Permissions beyond the scope of this public license are available at www.spikesorting.com.

Under the following conditions:

- You must attribute this work referring to the authors (Oliynyk A., Bonifazzi C., Montani F. and Fadiga L.) and citing the source URL.
- You may not use this work for commercial purposes*.
- You may not alter, transform, or build upon this work.

2. NO signal acquisition features or on-line classification functionality of FSPS^{TM} are limited under CCPL BY-NC-ND.

3. The software is a biomedical tool that should only be used by, or under the direct supervision of, an experienced researcher or licensed practitioner.

4. This software should not be utilized without careful review of the complete instruction manual, provided in the application folder or available at the web address: http://www.spikesorting.com/documentation.aspx.

5. Periodic updates for the software may become available, and the user accepts their obligation to register any changes in their e-mail address at www.spikesorting.com (if registered) should they wish to be informed about new releases. Users are also urged to check the www.spikesorting.com website for announcements of software upgrades.

6. LIMITATION OF LIABILITY. TO THE FULL EXTENT PERMITTED BY APPLICABLE LAW, THE LICENSEE RECOGNIZES AND ACCEPTS THAT: A) THE SOFTWARE IS SUPPLIED "AS IS," AND THE AUTHOR DOES NOT GUARANTEE THAT THE SOFTWARE IS FREE OF BUGS, FLAWS OR DEFECTS; B) THE AUTHOR DOES NOT GUARANTEE THAT THE SOFTWARE MEETS THE SPECIFIC NEEDS OF THE USER; AND C) THE AUTHOR EXPRESSLY DISCLAIMS ANY RESPONSIBILITY FOR ANY

DAMAGE, LOSS OF PROFITS, LOSS OF BUSINESS, LOSS OF DATA, DAMAGE TO COMPUTERS OR PERIPHERAL EQUIPMENT, OR ANY OTHER DIRECT, INDIRECT, INCIDENTAL, CONSEQUENTIAL, SPECIAL OR PUNITIVE DAMAGE, OR ANY LOSSES OF ANY KIND, ARISING DIRECTLY OR INDIRECTLY FROM THE USE OF THE SOFTWARE, CAUSED TO THE USER, PATIENT OR ANY THIRD PARTY. THE FOREGOING LIMITATIONS APPLY EVEN IF THE SOFTWARE CEASES TO FULFIL ITS ORIGINAL AND INTENDED PURPOSE.

7. The author does not guarantee the user any form of training, support or instruction regarding use of the software.

By installing FSPS™ you agree to the terms and conditions listed above.

Author:
Dr. Andriy Oliynyk
Section of Human Physiology,
Department of Biomedical Science and Advanced Therapy,
University of Ferrara,
Via Fossato di Mortara 17-19,
44121 Ferrara (FE), Italy

* - To obtain a Commercial License please delete CCPL (file 'license.png') manually, run the software and provide us with an authorization code necessary for your individual Commercial License. Please, allow up to 48 hours for an activation file to be delivered. USB security key will be released and delivered after FSPS™ Commercial License purchase.

Technical Requirements

/Minimum hardware, software and operating system specifications/

Hardware

1. National Instruments I/O Digital Acquisition Board*, having at least:
   - ✓ 1 Analogue input channel (sample rate 10 kS/S) with a Measurement Type – Voltage.

2. Computer**
   - ✓ Processor-form factor: Pentium II or above.
   - ✓ RAM: 1 GB or more.
   - ✓ Display: at least 1280 x 800 pixel resolution (1280 x 1024 is recommended).
   - ✓ Video Card: 128 Mb / 24 bit-colours / resolution 1024 x 800 or higher.
   - ✓ Monitor: 15 inch / resolution 1024 x 800 or higher (1280 x 1024 mode is recommended).
   - ✓ Hard Disk (drivers free): 500 Mb of free space.

3. Software
   - ✓ Windows XP / 2000 / Vista / Vista x64 / 7 / 7 x64 /
   - ✓ NI-DAQmx drivers (free at www.ni.com)
   - ✓ LabVIEW™ Run-Time Engine 2009 (free at www.ni.com)

---

* for new data acquisitions or online classification only.
** some netbooks running Windows XP or Windows 7 are also supported.

Installation

The installation process is performed as follows:

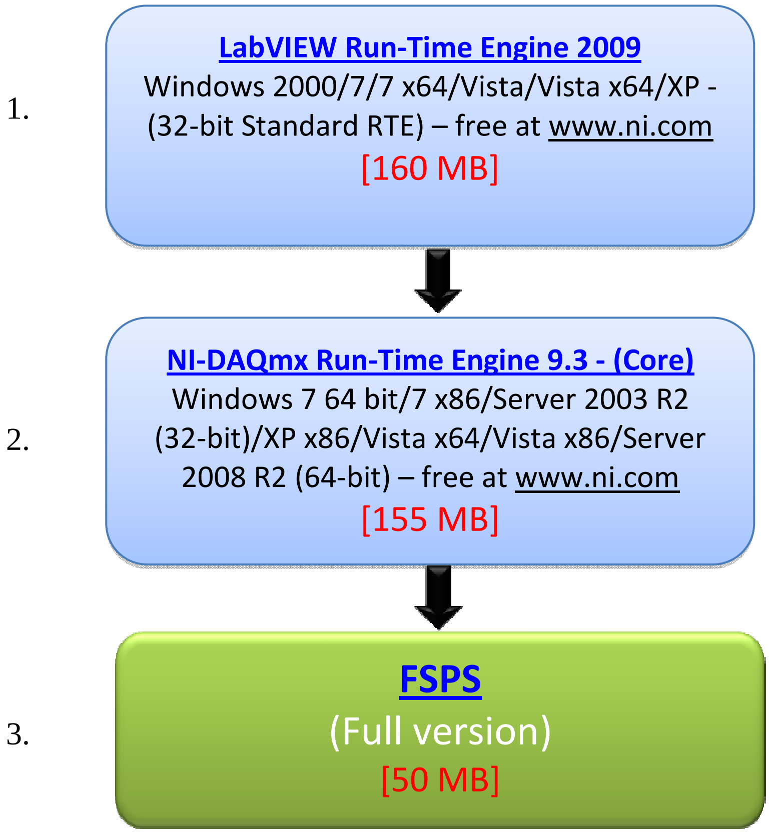

---

1. First, install **LabVIEW™ Run-Time Engine 2009 - Windows 2000/7/7 x64/Vista/Vista x64/XP - (32-bit Standard RTE)** – Installation file "LVRTE90std.exe" [160 MB] provided to registered users free of charge by NI (www.ni.com). Alternatively, it can be downloaded from http://www.spikesorting.com (*Download* page).

2. Next, install DAQmx device drivers supplied together with your NI DAQ device (check at www.ni.com for details). If you don't have NI DAQ drivers installed, even if you intend to use FSPS™ software exclusively for off-line analysis (without acquisition board), you should nevertheless proceed with installation of **NI-DAQmx Run-Time Engine 9.3 - (Core)**. Installation file "NIDAQ930f2Core.zip" [155 MB] is provided to registered users free of charge by NI (www.ni.com). Alternatively, it can be downloaded from http://www.spikesorting.com (*Download* page).

3. Install FSPS™ program: http://www.spikesorting.com [approx. 50 MB]. (*Download* page).

Uninstall notes:

Use Uninstall FSPS™ (file "unins000.exe"), located in the FSPS application folder, to remove the software from computer. To uninstall LabVIEW™ Run-Time Engine 2009 and NI Device Drivers you should use the standard tool on the Windows Control Panel.

Chapter 1. Getting Started

With NI Digital Acquisition Board (NI DAQ)

To use FSPS^{TM} with a NI DAQ, connect the latter to the computer and configure your NI DAQ device as specified in the relevant manual before running the FSPS^{TM} programme.

Next run the programme "**fsps.exe**" from the FSPS^{TM} destination application folder ("C:\Program Files\fsps.exe" by default).

As soon as the programme starts, configure your NI DAQ board. To do this, activate the DAQ control button at the **DAQ** tab (Control Tab 1) and set up I/O parameters against your particular board configuration (Fig. 1a).

/Figure 1a/

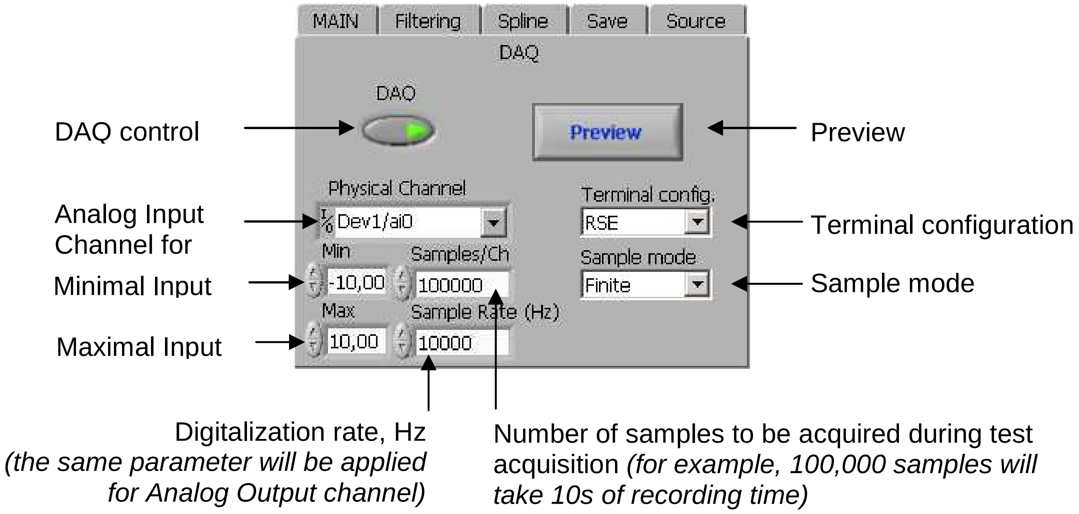

Press Preview button to view the signal at the selected channel in separate window (Fig. 1b).

/Figure 1b/

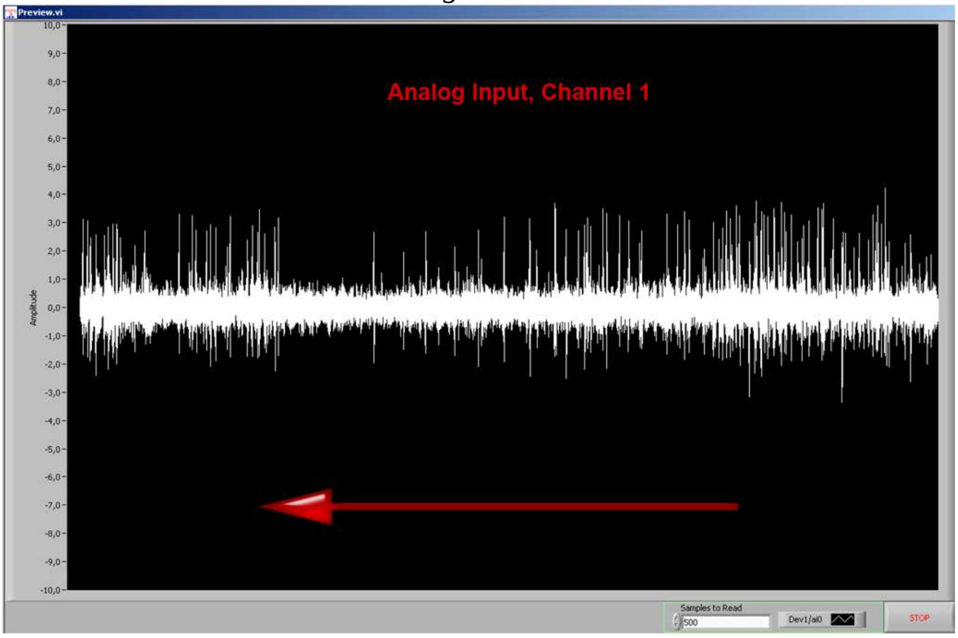

Without NI Digital Acquisition Board

If you don't have an NI DAQ connected or wish to use FSPS™ software solely to analyse existing recordings (offline), you should switch OFF the DAQ control button (default value) right after running the program (Fig. 1c). Thus, further operations and analysis will be performed on existing recordings selected in the **Source** tab.

/Figure 1c/

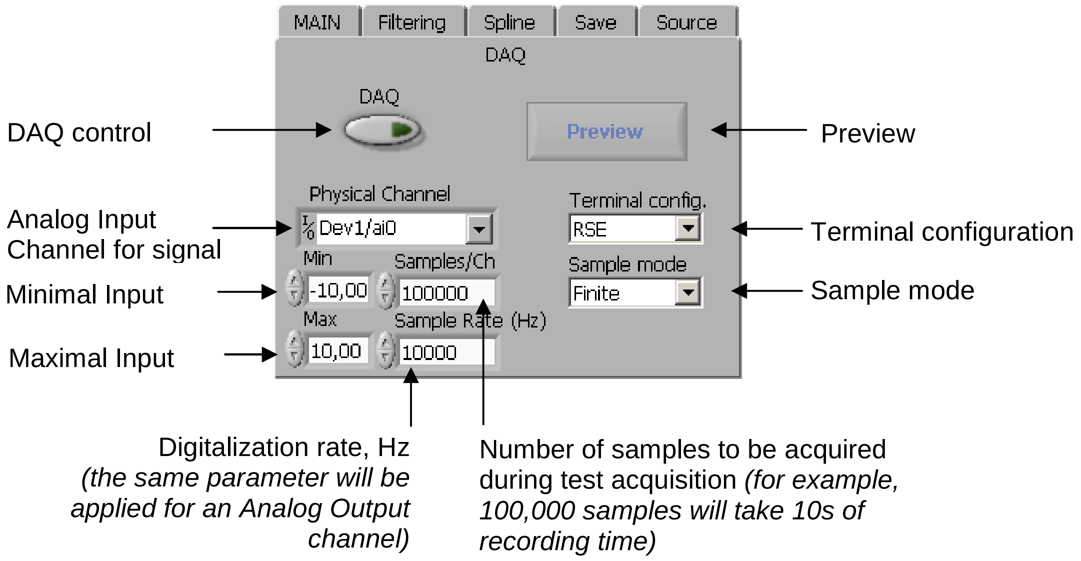

Chapter 2. Continuous Signal Acquisition and Off-line Spike Sorting

/Figure 2/

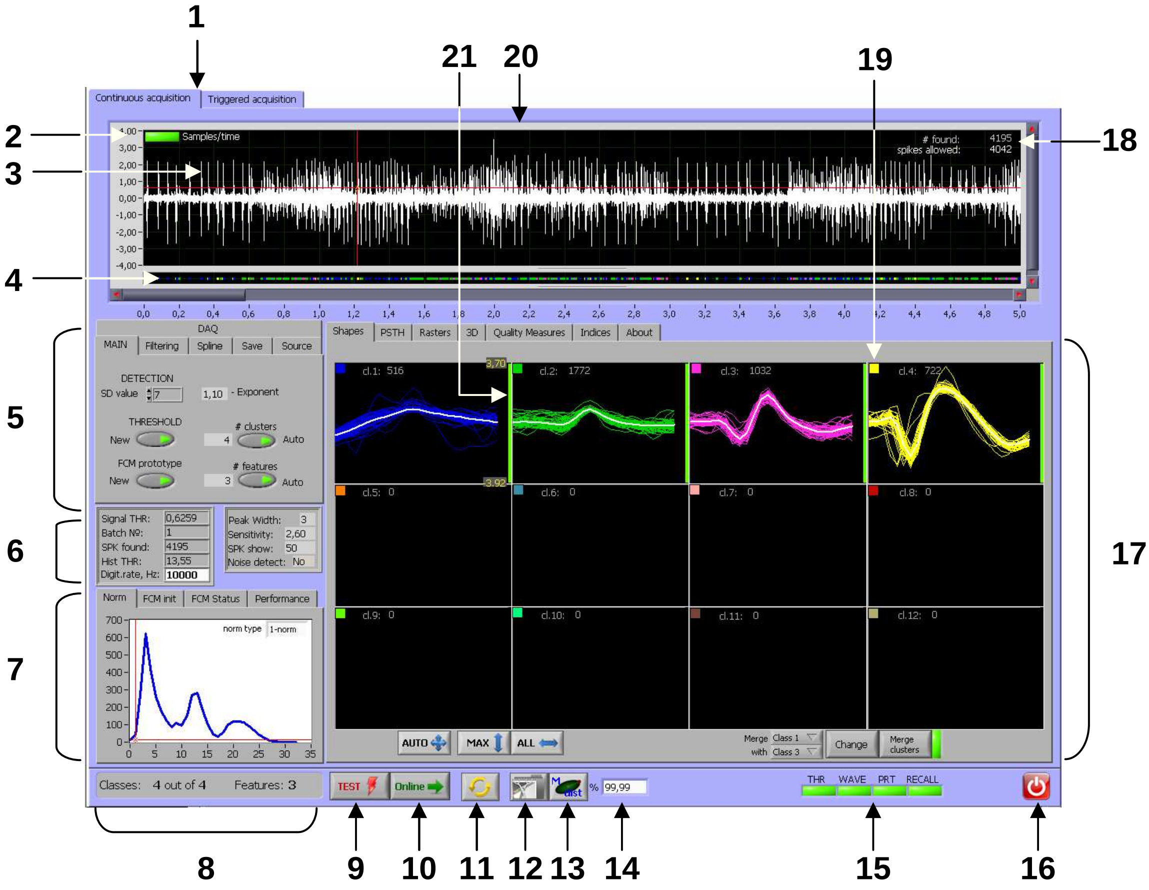

1 - ***Control Tab 0*** switches between Continuous and Triggered Acquisition.

2 - Control switch for X-axis scale (samples or absolute time is used in graphs and PSTH).

3 - Raw signal graph.

4 - Spike locator (the colour of the dot corresponds to the colour of the spike shape in 13).

5 - ***Control Tab 1*** *(selection of input parameters);* see Chapter 2.1 for details.

6 - Group automation indicators:

Signal THR – the voltage threshold level for the spike detection algorithm;

Batch № – the number of the data chunk that is currently being processed;

SPK found – the total number of spikes detected in the data set being processed;

Hist THR – the level to threshold *norm* histogram (needed to determine the number of classes);

Digitalization rate – is an important parameter indicating the number of samples per second of your acquired data. Since this parameter influences many algorithms of data analysis and visualization, we recommend paying particular attention to it.

Peak width – specifies the number of consecutive data points used in the least-squares fit for finding peaks in the *norm* histogram;

Sensitivity – an important parameter for adjusting the construction of the *norm* histogram, thereby influencing the number of clusters determined. Tolerated range is 1.8-3.3 (Default is 2.6, the most common for various types of signals and acquisitions);

SPK show – specifies the number of spike shapes to be visualized in each window of the Shapes Tab indicator;

Noise detect – indicates if the detection of noisy spike shapes based on Mahalanobis distance evaluation is *On* or *Off*.

7 - ***Control Tab 2*** *(FCM status and performance information);* see Chapter 2.2 for details.

8 - Information panel for number of clusters and features used.

9 - Control button TEST to RUN clustering/classification algorithm.

10 - Control button Online to RUN online classifier.

11 - Control button Redraw to update several on-screen parameters .

12 - Save button performs screenshots capture of spike-sorting results (currently visualized area of *Control Tab 3* is captured and automatically saved to the same disk space where data files are stored).

13 - Control button M-dist applies Mahalanobis Distance measurement to evaluate noisy spikes (actually, their PC scores) located in the multidimensional feature space, away from any class centre, using probability density (*see p.14*).

14 - Confidence interval for the Mahalanobis distance measurement.

15 - Group indicator of data processing.

16 - Control button to stop the program.

17 - ***Control Tab 3*** *(visualization and statistics);* See chapter 2.3 for details

18 - Information about detected and accepted spikes.

19 - Squares at the top left corner of each graph are controls to change the colour of the class.

20 - Sliding bar to navigate "Raw signal graph" ("**2**").

21 - The indicator of the cluster's quality (colour scale).

Chapter 2.1 (Control Tab 0, *Continuous or Triggered acquisition***)**

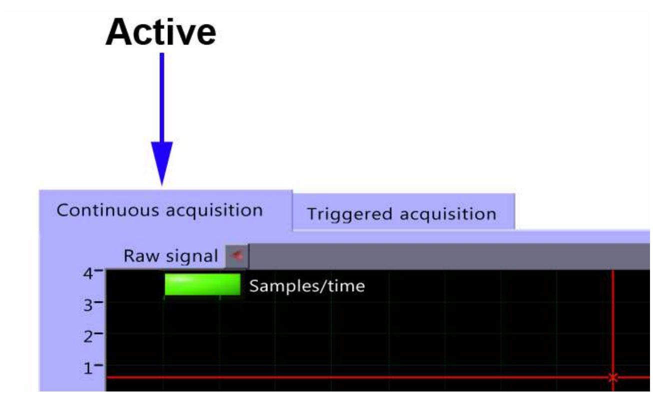

Allows you to choose between continuous (this chapter) and triggered (Chapter 3) acquisition, depending on your needs and experimental design.

Chapter 2.2 (**Control Tab 1,** *basic I/O parameters*)

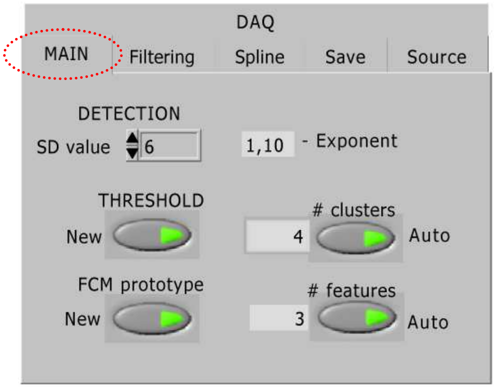

Main **Tab options:**

- "Detection" (SD value). Threshold level is estimated as:
  Thr = $SD_{value} \cdot \sigma_{noise}$, where σ_{noise} is an estimate of the standard deviation of the background noise. SD_{value} can vary from 4 to 8. Default SD_{value} is 7.
- THRESHOLD (New/Keep) is the control button allowing automatic (New) or manual (Keep) estimation of the threshold. In the latter case you can adjust it by moving the cursor (red line) inside the "Raw signal graph" (3).
- FCM prototype (New/Keep) allows the FCM clustering technique to make a new space segmentation and calculation of clusters' centres (New), which is a necessary step of FCM unsupervised clustering technique. "Keep" mode permits the classification of new objects and their membership values to the different classes for the given cluster centres, which is used during classification procedure.
- # clusters (Auto/Manual) allows automatic determination of the number of clusters to be counted (Auto). "Manual" operation forces clustering algorithm to find exact (indicated at the left side) number of clusters. The range is from 1 to 12.
- # features (Auto/Manual) allows automatic determination of the number of PCs to be used during clustering/classification (Auto). "Manual" operation allows user to manually choose dimensionality by inserting the value in the field next to the button. Range is 1 to 5.
- Exponent determines the degree of fuzziness of the resulting clustering process. As $m \to 1.1$ clustering tends to be fuzzy, while $m \to \infty$ results in crisp clustering. 1.1 is the default value.

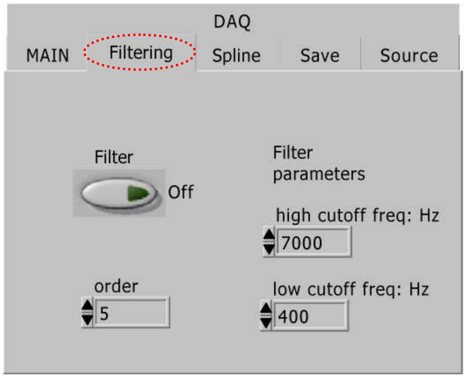

Filtering **Tab options:**

Filter (On/Off) is activating/deactivating software bandpass Butterworth filter. The default is Off.

- "high cutoff freq: Hz" is the high cut-off frequency in Hz. It must be greater than low cut-off frequency and fulfil the Nyquist criterion.
- "low cutoff freq: Hz" is the low cut-off frequency in Hz and must fulfil the Nyquist criterion. If low cut-off frequency is less than or equal to 0, or greater than half the sampling frequency value, the algorithm is not working. The low cut-off frequency must be less than the high cut-off frequency.
- "order" specifies the filter order and must be greater than 0. The default is 5. If the order is less than or equal to 0, the algorithm signals an error.

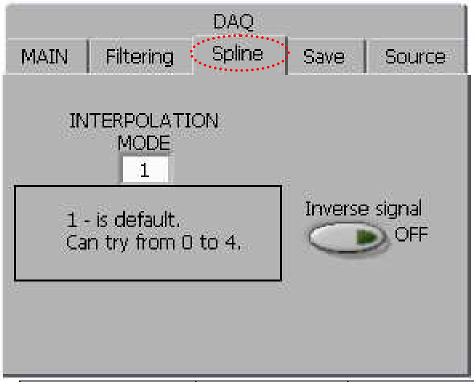

Spline Tab options:

- "INTERPOLATION MODE" (can be 0, 1, 2, 3 or 4). The default is 1. It determines slightly different strategies of waveform extractions and their alignment (depending on spike duration, acquisition rate etc.). Detailed information is indicated in the table below:

Interpolation mode	Length of extracted waveform, samples	Location of the peak, sample	Interpolation rate, times	Overall length of interpolated waveform, samples	Cut out samples, beginning/end	Length of resulting waveform, samples
0	12	4	3	36	6/6	24
1	18	7	2	36	6/6	24
2	36	12	2	72	4/4	64
3	36	14	1	36	6/6	24
4	72	24	1	72	4/4	64

- "Inverse signal" inverts the signal (ON); OFF, unchanged, is default.

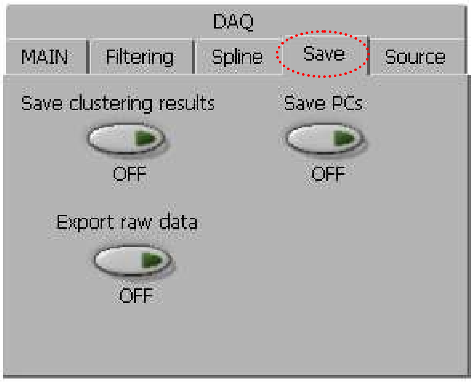

Save Tab options:

- Save clustering results control button allows the results of clustering/classification to be saved automatically (when ON). At the end of each run of clustering/classification algorithm (by pressing the TEST button) it saves a text file (*.tcs) in the same folder. The file contains:
1) Peak occurrence time (1st value in each row);
2) Class label (2nd value in each row)
3) 3rd – the end of each row is an interpolated waveform of the spike

Therefore, the number of rows corresponds to a number of extracted spikes, while the number of columns depends on the length of the waveform (consult the table above).

- Save PCs control button allows Principal Components to be saved automatically (when ON). At the end of each run of clustering/classification algorithm (by pressing the TEST button) it saves a text file (*.psvd) in the same folder. The file contains:
1) First column - Class label (2nd value in each row)
2) Second column till the last one - Principal components (like PC1, PC2, PC3 … PCk).

- Export raw data control button allows to save your current channel (visualized in "Raw signal graph", p.3 at the Figure 2) in separate .txt file.

Source Tab options:

Folder path (use dialog) is a path control that allows you to select a file/folder with data. Before selecting the folder you have to select the file type from drop down menu. At the moment there are 6 different formats available:

a) Binary (GE), a stream of unsigned 16-bit integers (U16) having **little-endian** byte order. There is no header information.

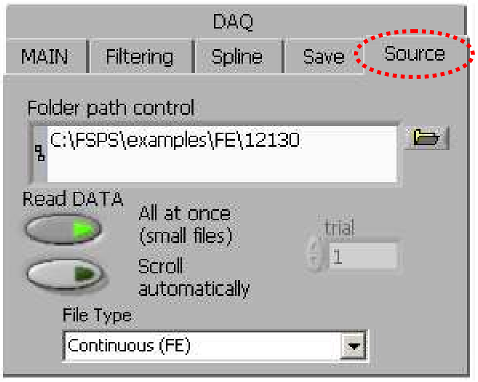

b) Text (UD, TXT) for text files as follows:
X,Y.YY,
where X – is a progressive number of samples;
,- to delimit fields in the text file
Y.YY – is a value in which the dot is used as the decimal separator (see example below):

1,-5.20
2,-7.40
3,-6.97
4,-4.57
5,-1.67
6,-1.05
etc.

c) Triggered (FE) reads data from the 16-bit Integer file, which contains 12 rows corresponding to the triggered acquisition trials.

d) Binary (MAP) reads .map files generated by the AlphaMap software from Alpha Omega (Nazareth, Israel). This converter is experimental.

e) Simulated (Qui) – another kind of TEXT file to import data saved in engineering format as follows:

```
-5.2651722249719632e-002
-3.1241866532139659e-002
-2.8216166649551004e-003
 2.7610470619340344e-002
 9.4321581225495860e-002
 1.0073366268315526e-001
```
…
The data organized in this way can be found here: http://www.spikesorting.com/simulated-data.aspx. They are the same simulation datasets used by R. Quian *Quiroga*, Z. Nadasdy and Y. Ben-Shaul. Unsupervised Spike Detection and Sorting with Wavelets and Superparamagnetic Clustering // Neural Computation 16, 1661–1687 (2004).

f) Continuous (FE)* – this is the format that uses FSPS™ software for continuous data acquisition ("HDD streaming" button). The file has 8032 bytes (1004 I64 values) header information. First four values in the header are:

1. The number of channels (depends on how many input physical channels are selected in the "I channel" control).
2. The number of samples in one data chunk for each channel (1000 is a default).
3. The digitalization rate as samples/s (10000 is a default).
4. The number of reserved U8 lines (the default is 0). At the moment, the rest 1000 I64 values reserved for multi-electrode array and brain-computer interface (*in progress*).

After the header comes a series of chunks. A chunk consists of four parts:
- length (integer I32);
- eight U8 values (can be “0” or “1”) for digital events (if any);
- I32 integer of millisecond timer value that has been split into its component bytes (four U8 values).
- an array of single-precision (SGL) elements of measured data (multiple of a number of samples in one data chunk for each channel, see above).

* - if you need just export the data recorded with FSPS™ software into your software you may use “Export raw data” at the **Save Tab**. It saves your current channel in the .txt file without the need to build your own file converter.

- READ DATA – there are two additional buttons that allow a specific part of the binary file (designed primarily for long GE files) to be read, allowing it to be processed in batches. The number of such batches is counted automatically when this option is selected. For triggered (FE) data the same buttons allow solely a specific trial to be read.

Note that for batch processing of long files it is necessary to switch OFF all automatic options at the **Main Tab** to maintain the same parameters for all parts of the file. The report (if you want generate and save it using **Save Tab**) will show information from these batches as one continuous file.

Chapter 2.3 (**Control Tab 2,** *FCM status and performance information*)

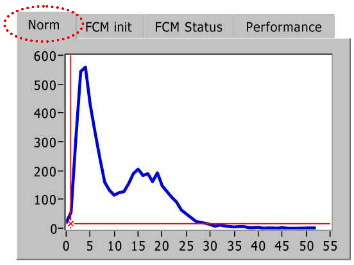

Norm Tab **contents:**

A histogram showing the distribution of *norm* values for every score of PCs. The histogram is automatically thresholded (red horizontal line) to eliminate the noise content.

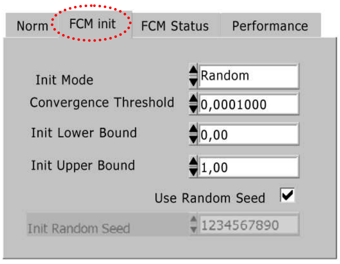

FCM init Tab **contents:**

Initial parameters of FCM clustering algorithm

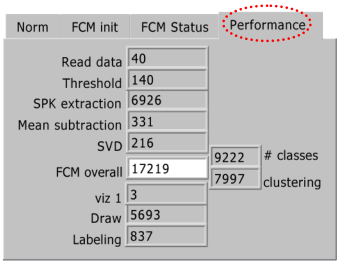

Performance Tab **contents:**

Information about the absolute time of each off-line running procedure in milliseconds.

Chapter 2.4 (Control Tab 3, *Visualization and Statistics*)

Shapes Tab contents:

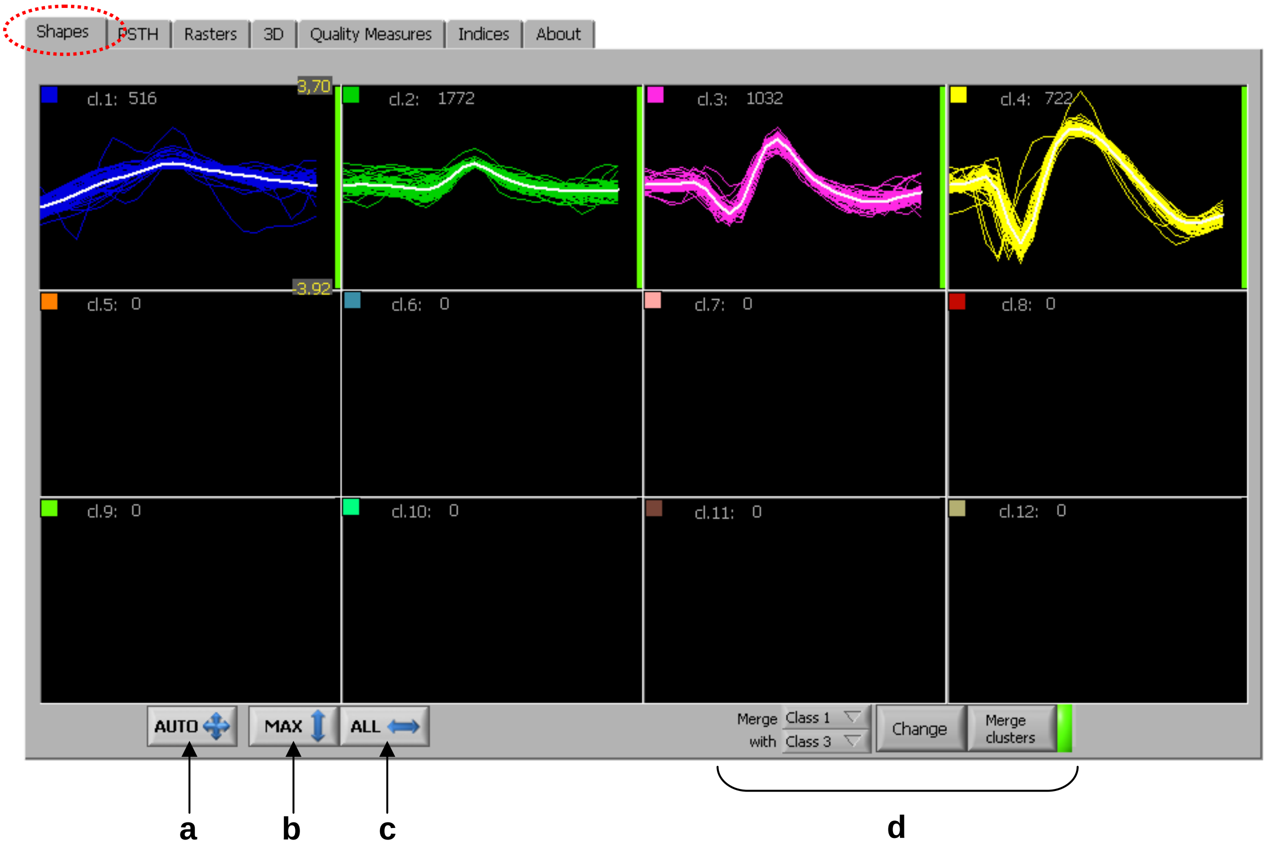

The number of spikes shown for each cluster can be adjusted using the tool SPK show, found on control window "**6**" (see Figure 2); the default is 50. The white spike profile is an average of that class. AUTO button "**a**" auto-scales (X and Y axes) the first window (shapes marked in blue in our specific case). MAX button "**b**" performs the same operation as "**a**", but takes into account the highest amplitude found in any of the classes shown. Clicking ALL button "**c**" applies the scales and ranges selected for the first window to windows 2-12.
You can also adjust Y scale parameters manually by entering its numerical values in the first window (click ALL button "**c**" to apply it to all of the other windows).
Command group "**d**" allows you to change the order of the classes manually (by clicking on button Change) or merge one class with another (by clicking on the button Merge clusters). To return to the initial cluster and order, switch OFF green LED located to the right of the Merge clusters switch.

PSTH Tab contents:

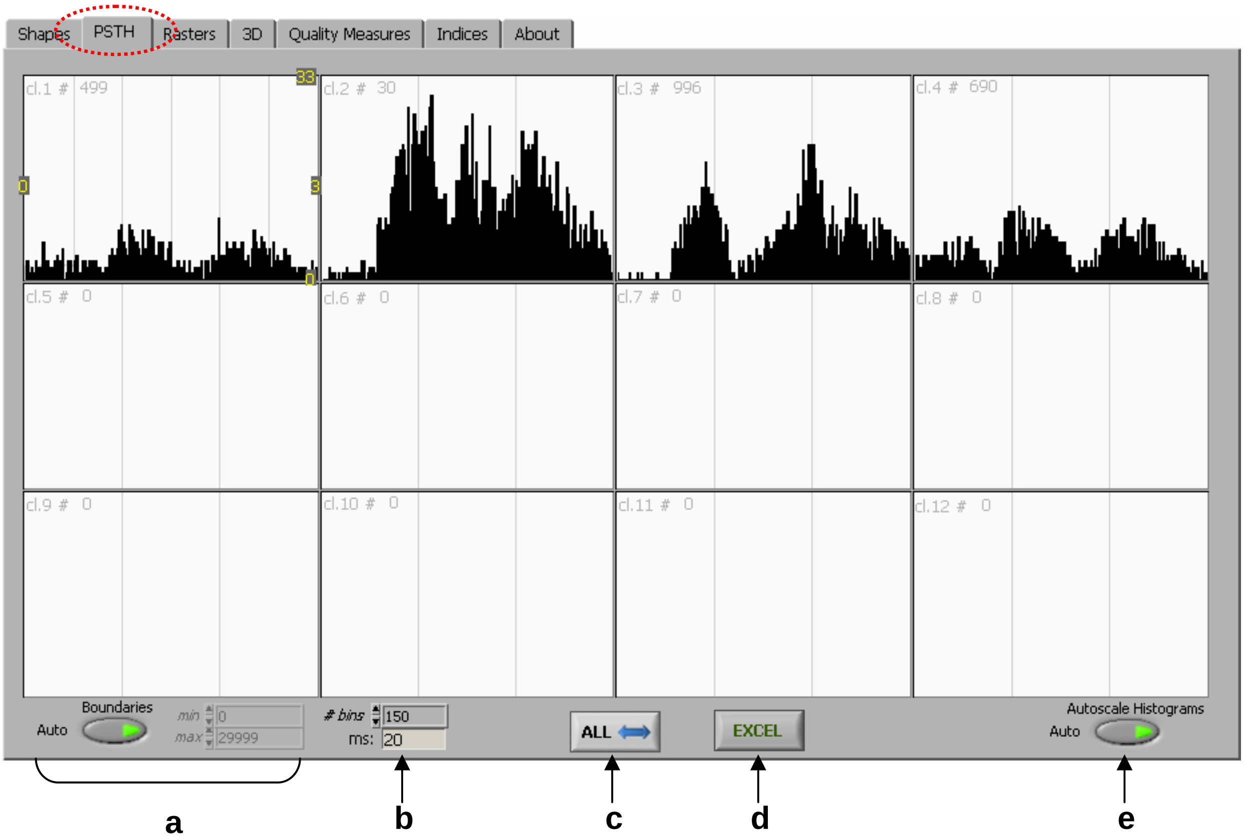

PSTH histograms are particularly useful in triggered trials but can also be used in continuous acquisitions. Histogram length and height can be set up automatically, when "**a**" and "**e**" controls are "ON" (*default* settings), or manually (controls "**a**" and "**e**").

Use *Switch 2* (see Figure 2) to change X scale between samples or seconds. Both X and Y scales can be set manually.

To change the duration of the ***bin,*** select the appropriate "**# *bin*"** parameter (control "**b**").

ALL button "**c**" extends the first graph's settings to the other graphs displayed, as described above for the **Shapes Tab.**

Detailed information about the contents of each ***bin*** for each histogram can be obtained by pressing the Excel button ("**d**"). This exports the information to Excel tables, one for each class. Note: Microsoft Excel must be installed for this to function.

Rasters contents:

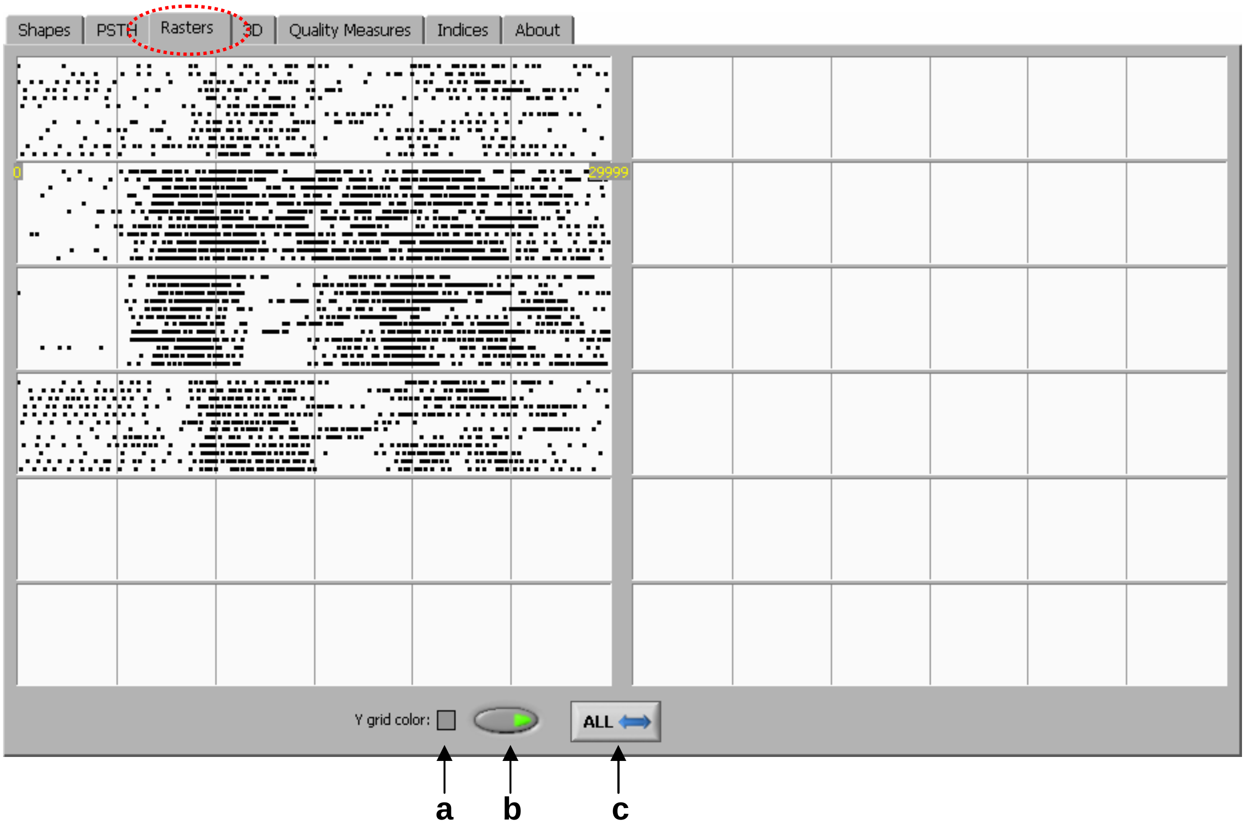

Displays Raster plots for each detected class and trail. Button "**b**" switches ON and OFF vertical grid. Grid colour can be chosen by clicking on "**a**". ALL button "**c**" function is the same as described above for the **Shapes** **Tab.**

3D Tab contents:

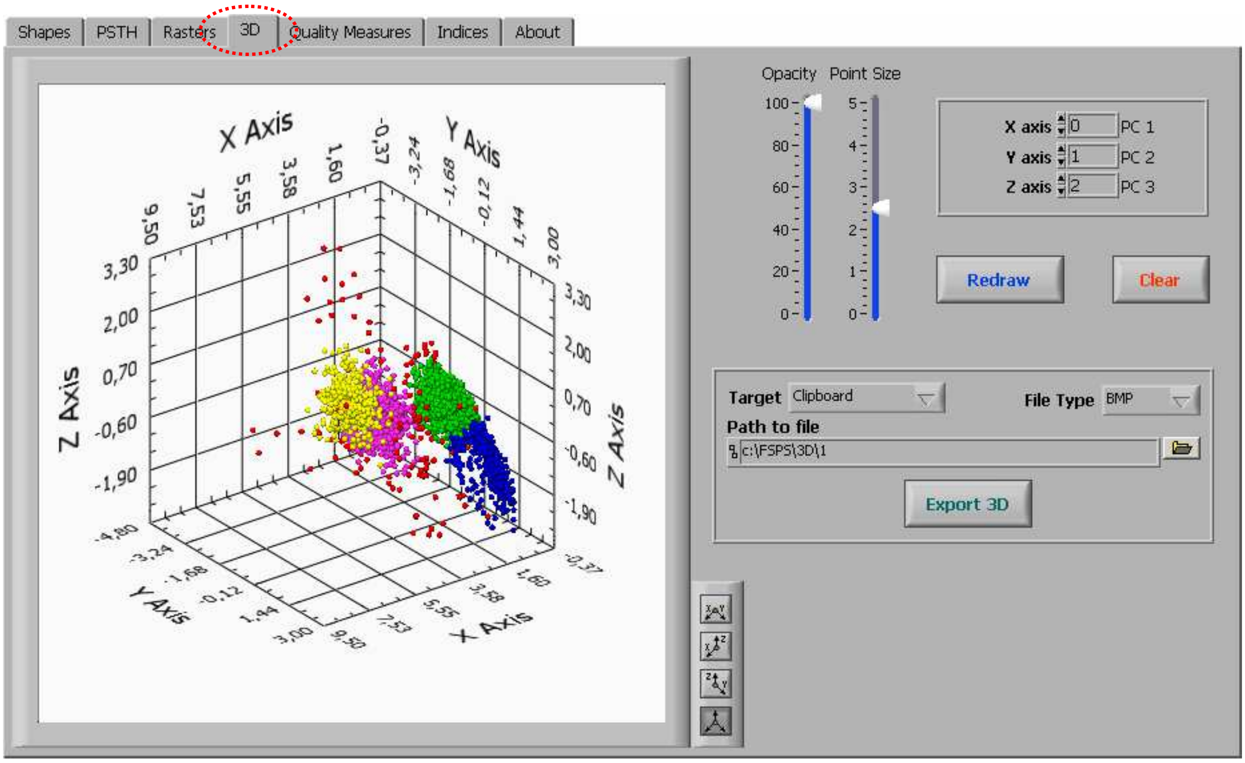

Shows the 3D Scatter Plot of the first three PCs accumulating maximal variance. Other PCs can be viewed by selecting their numbers using the controls in the top right corner.

Other graph properties:
1) Click and drag the graph to change the view direction.
2) Press the **<Shift>** key when you drag the mouse up to zoom in and down to zoom out.
3) Press **<Ctrl>** key when you drag the mouse to move the graph around.
4) Change the view direction mode using the projection palette (to the lower right of graph).

Quality Measures Tab contents:

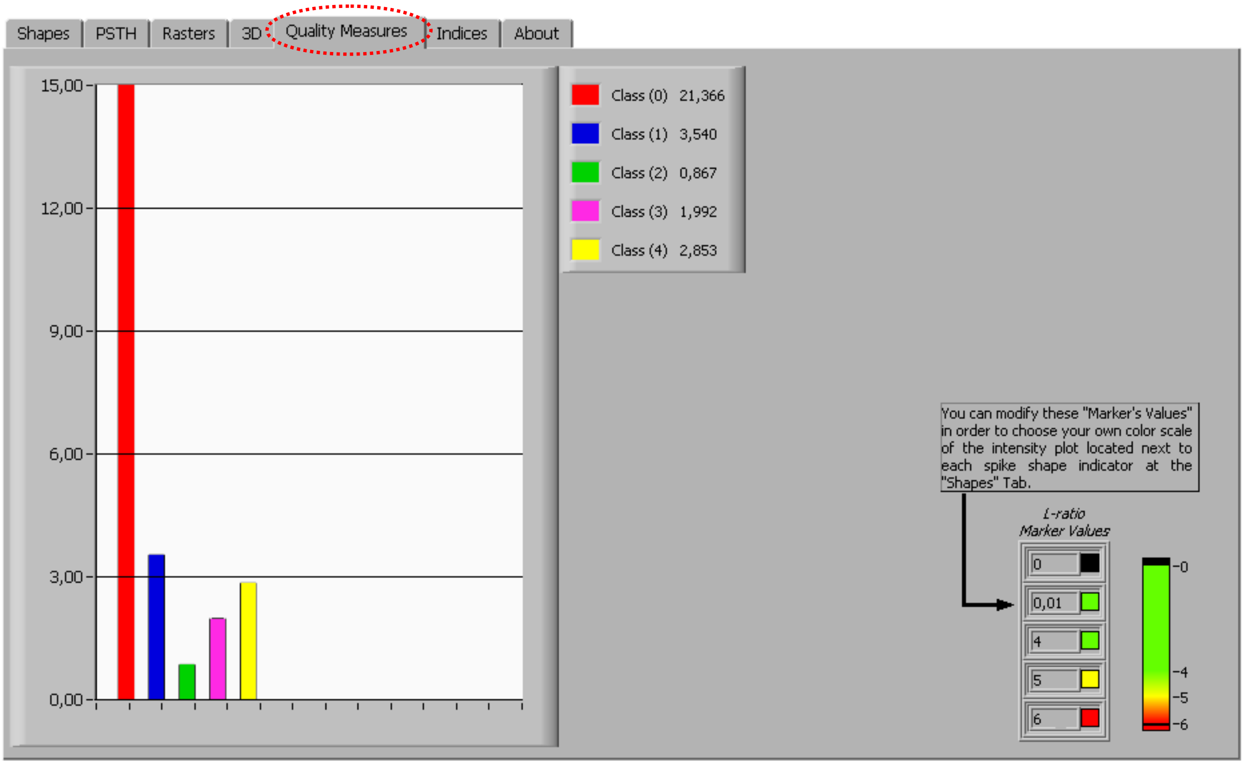

Any kind of clustering or classification needs an objective measure of its quality the most conventional indices associated with FCM, including *partition coefficient*, *partition entropy* and *proportion exponent*, are incorporated in the Toolbox, although our tests show that the index L_{ratio} is superior and so is the default quality measure. In the vast majority of cases, values below 5 (threshold) means acceptable cluster quality. Values greater than 5 are usually associated with poor clusters (with reference to their compactness and distance from other clusters in the dataset).

Exact L_{ratio} values for each cluster are shown in the graph legend.

For more details see: doi:10.1016/j.neuroscience.2004.09.066

Indices Tab contents:

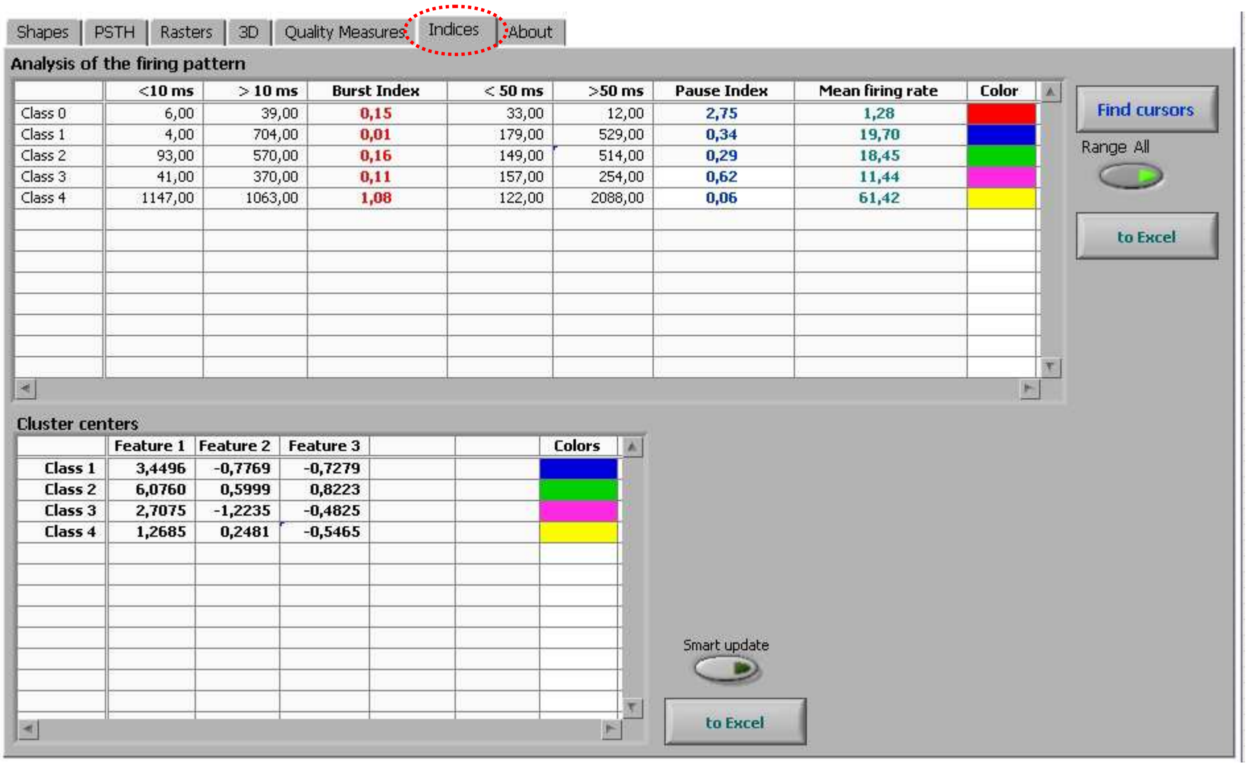

	<10 ms	> 10 ms	Burst Index	< 50 ms	>50 ms	Pause Index	Mean firing rate	Color
Class 0	6,00	39,00	0,15	33,00	12,00	2,75	1,28	
Class 1	4,00	704,00	0,01	179,00	529,00	0,34	19,70	
Class 2	93,00	570,00	0,16	149,00	514,00	0,29	18,45	
Class 3	41,00	370,00	0,11	157,00	254,00	0,62	11,44	
Class 4	1147,00	1063,00	1,08	122,00	2088,00	0,06	61,42	

	Feature 1	Feature 2	Feature 3	Colors
Class 1	3,4496	-0,7769	-0,7279	
Class 2	6,0760	0,5999	0,8223	
Class 3	2,7075	-1,2235	-0,4825	
Class 4	1,2685	0,2481	-0,5465	

Burst Index (BI) defined as the number of ISI < 10 ms divided by the number of ISI > 10 ms.
Pause index (PI) defined as the number of ISI > 50 ms divided by the number of ISI < 50 ms.
For more details see: doi:10.1016/S0090-3019(99)00030-0

The table below shows the coordinates of cluster centers.

The button Smart update is experimental. It allows tracking of clusters in the feature space when parameters of spike waveform is changing due to some deterministic (non-noise) influences (e.g. small periodic displacements of the location of electrical activity relative to the microelectrode, systematic reduction of the amplitude of an action potential when the cell fires in a sustained high-frequency burst, systematic slow drift of electrode etc).

About (your license info):

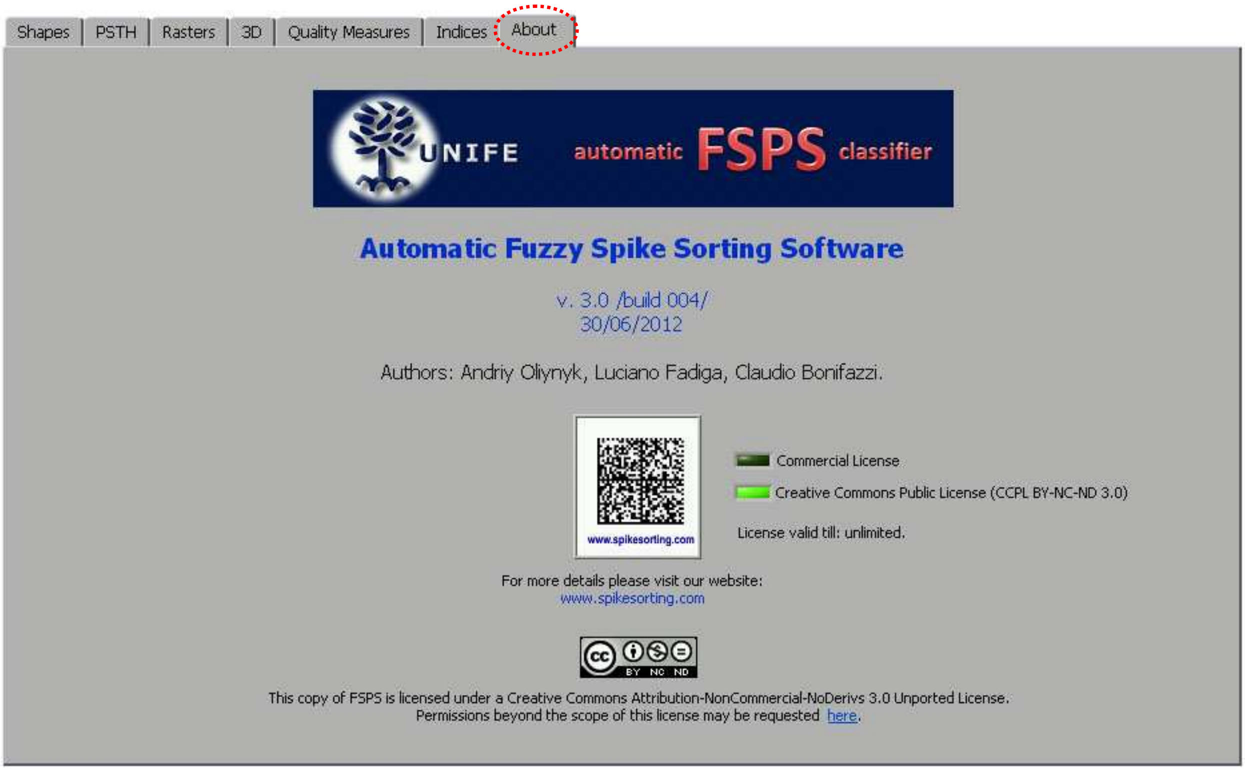

Please refer to this page to check your License. The DataMatrix image confirms the presence of the License file, while the green indicator below shows its type and validity.

Note that the Creative Commons Public License (CCPL BY-NC-ND) for academic non-commercial use is included in the installation package.

Chapter 3. Triggered Acquisition and Off-line Spike Sorting

/Figure 3/

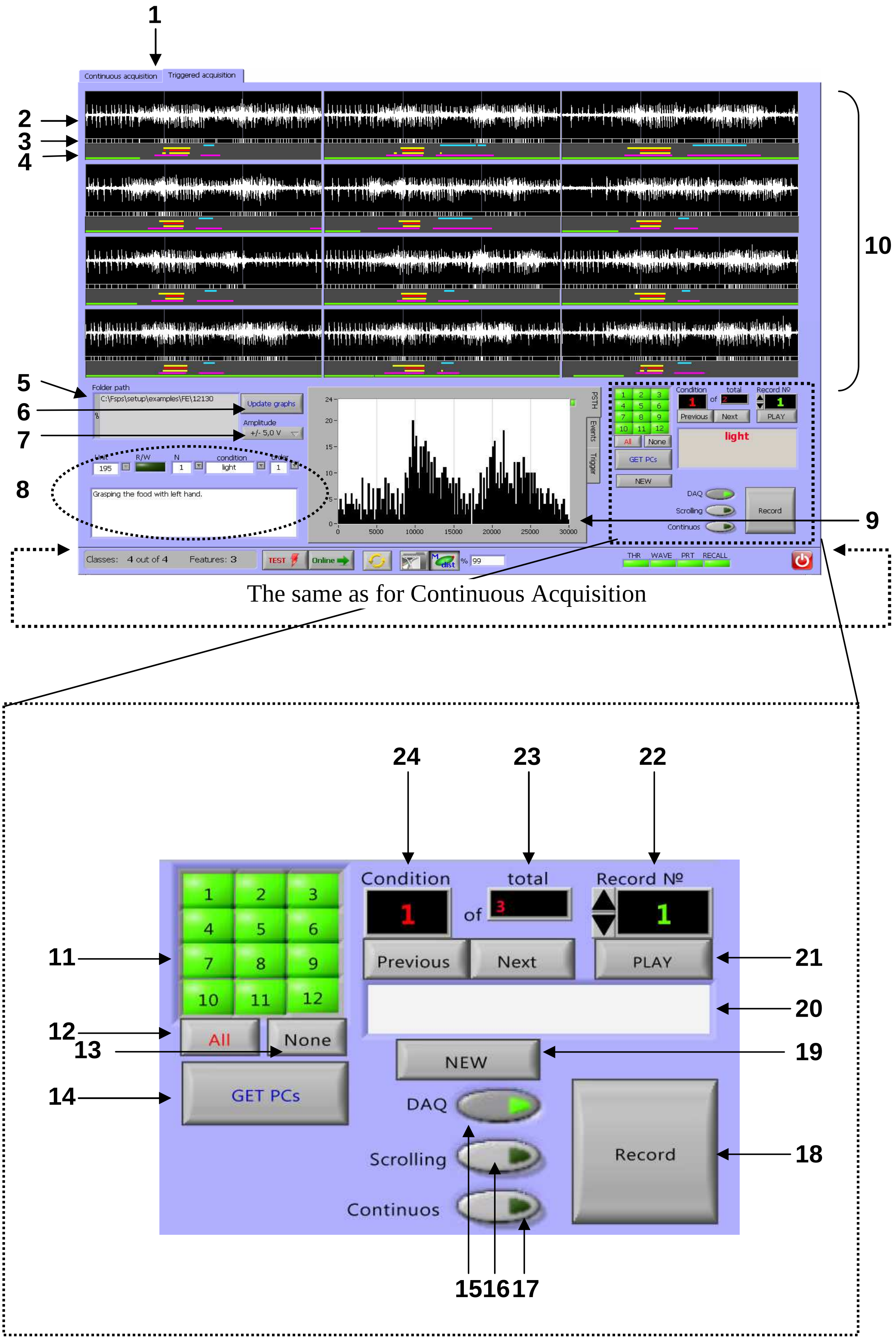

Legend:

1 - External Tabs (Tab control 0) switch between Continuous and Triggered acquisition.

2 - Raw signal graph (trial 1 of 12).

3 - Spike location (spikes are determined by hardware voltage discriminator external to the FSPS™ programme).

4 - Coloured lines correspond to different external events during task execution

5 - Name of the destination folder.

6 - Update graphs can be used to redraw raw signal graphs (2) when necessary

7 - Amplitude allows adjustment of Y scaling factor (for raw signal graphs, p.10) in pull-down menu.

8 - Group labelling information for each triggered acquisition set.

9 - Tab Control 1 (advanced triggering settings and PSTH, based on spike occurrence determined by hardware voltage discriminator).

10 - Group graphs and indicators representing a particular recording session (12 trials in this case).

11 - Group control to include trials for PSTH composition

12 - Fast selection of all trials to be included in hardware-based PSTH graph

13 - Fast deselection button for hardware-based PSTH graph

14 - Fast automatic extraction and visualization of PCs scores for each trial

15 - DAQ control enables triggered acquisitions and recording functions 16-19

16 - Scrolling control allows automatic adjustment of the “Record n°” and “Condition” (if many) when the acquisition of the current test is complete.

17 - Continuous control performs the same operation described below in 18, automatically incrementing “Record n°.” and starting a new trial. It deactivates when “Record n°” reaches a value of 12 and all your “Conditions” are complete.

18 - Record prepares to record trigger event (by default at PFI0 of NI DAQ), upon which it writes/overwrites acquired signal at the position indicated in control 22

19 - NEW allows the creation and design of your acquisition set by selecting and naming different experimental “Conditions”.

20 - Indicates the current Condition in the Set.

21 - Control button PLAY converts the raw signal of the current trial (indicated at “Record n°” control) into sound file (WAV format) and reproducing it through computer sound board.

22 - Record n° control serves to navigate between trials (write, overwrite or convert into WAV format). To select particular trial (to overwrite it, for instance) the Scrolling button must be switched “Off”.

23 - Indicates the total number of “Conditions” in the recording set.

24 - Indicator of the current “Condition”.

Chapter 3.1 (Control Tab 0, *Continuous or Triggered acquisition***)**

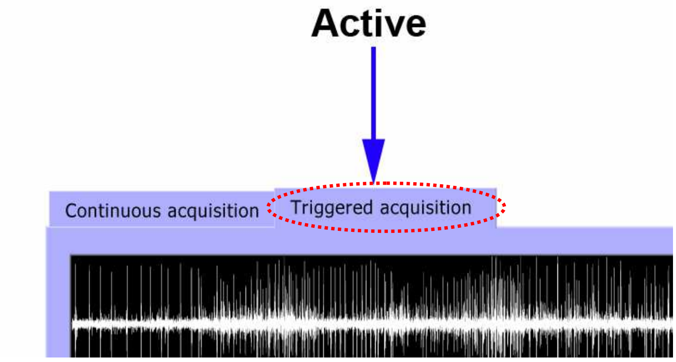

Allows you to choose between continuous (Chapter 2) and triggered (this chapter) acquisition depending on your needs and experimental design.

Chapter 3.2 (Control Tab 1, *hardware info & settings***)**

PSTH internal tab (hardware-based)

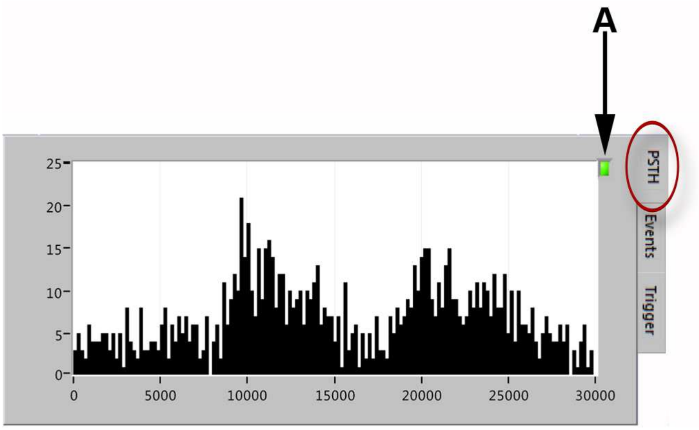

Commonly, electrophysiological recording systems contain an option to perform spike isolation using hardware voltage discriminator. This makes it possible to roughly characterize neuronal activity and its specificity instantly and during recording process. This function is also offered by this software, in parallel with the Fuzzy Spike Sorting procedure. Please note, that to acquire the signal from your window discriminator hardware (in our case it is channel 2), you need to reserve a separate I/O channel at your DAQ .

“**A**” can be used to switch auto scale for PSTH graph ON (green) or OFF (dark).

Events **internal tab** (supplemental TTLs)

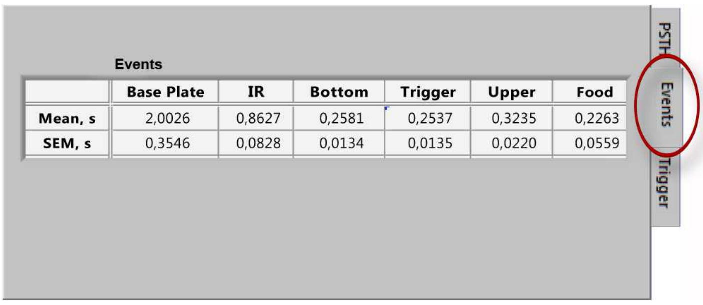

	Base Plate	IR	Bottom	Trigger	Upper	Food
Mean, s	2,0026	0,8627	0,2581	0,2537	0,3235	0,2263
SEM, s	0,3546	0,0828	0,0134	0,0135	0,0220	0,0559

The average duration (MEAN ± SEM) of each external event occurring during execution of the task is given at this Tab. Indeed, during your electrophysiological study (single unit recordings, or whatever), you may need to control some other external parameters, such as the duration of button or sensor holding, precise touch timing and/or kind of kinematic data etc., This additional information could help the experimenter to control task execution or to align recording neuronal activity to one of these external events.

The current version of the software supports up to different 6 TTL channels (thus 6 events); however their number depends mainly on the type of DAQ board utilized.

One example of their application in an example experimental task is shown below. The task in question was the execution of goal-directed movement by the left hand of the monkey during recording from premotor cortex (area F5) of the right cerebral hemisphere.

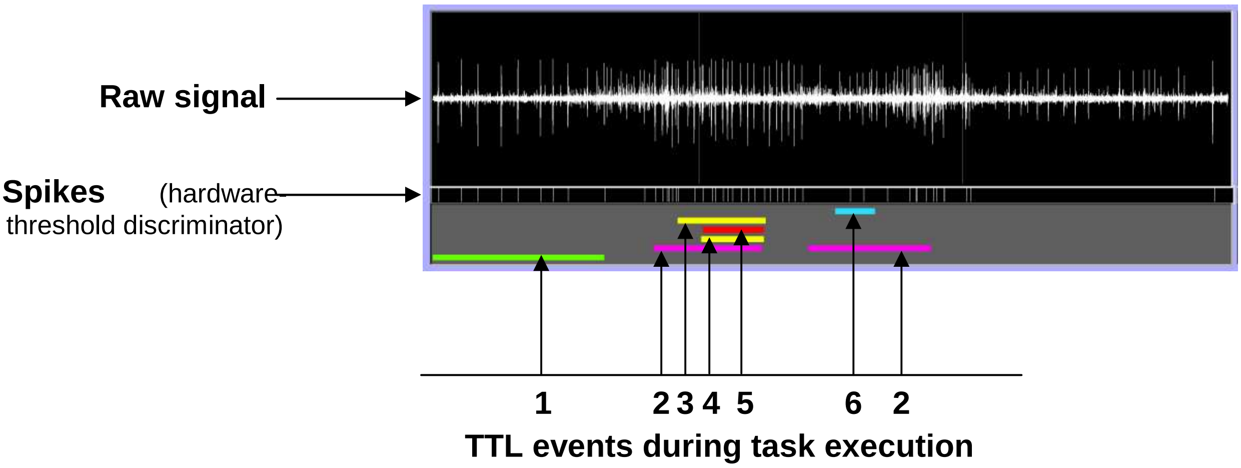

The duration of the trial was 3s. In this time the monkey reached out his hand to the handle of the small door of the box, opened it and took the food as a reward. The Figure shows, as well as the neuronal activity recorded by the electrode, the external events characterizing this behavioural task: "**1**" (green line) - the hand of the monkey is still in its initial position; "**2**" (pink) - the hand crosses the infra-red barrier in front of the door; "**3**" (upper yellow) - touching the upper sensor of the handle; "**4**" (lower yellow) – touching the lower sensor of the handle; "**5**" (red) - main trigger, contact with both the upper and lower sensors of the handle; "**6**" (blue) - taking the food from the box.

Trigger **internal tab** (hardware settings)

Before starting acquisition, use this Tab to define desired digitalization settings and trigger parameters.

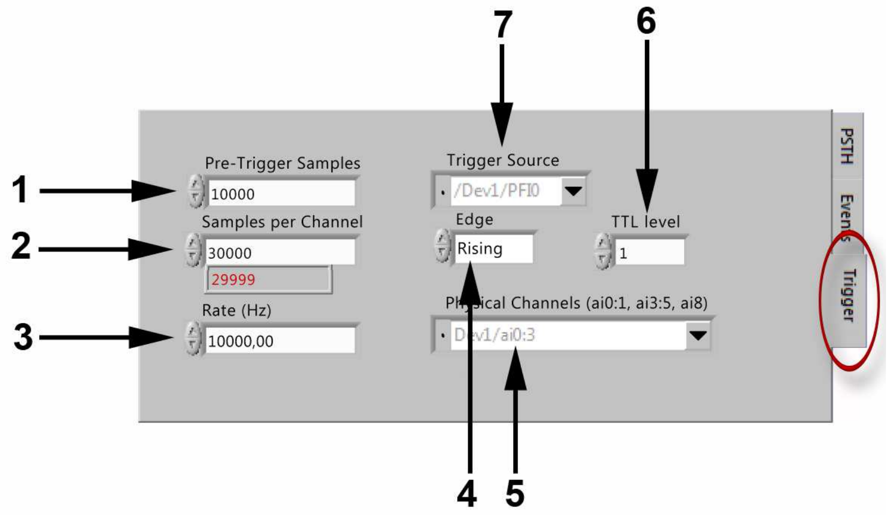

1 – The number of pre-triggered samples. In this specific case 10000 samples equal 1 s (Digitalization rate specified in 3 is 10000 samples/s);
2 – Total duration of the trial. In this case 30000 equals 3 s (Digitalization rate specified in 3 is 10000 samples/s);
3 – Digitalization rate specified (samples/s);
4 – Physical channels for event TTL signals;
5 – Direction of electrical edge for trigger;
6 – Adjustments for TTL level;
7 – Physical trigger channel.

Chapter 3.3 (Create New Set**)**

This interface window emerges after pressing the button New (“**19**” in Figure 3). It allows you to initiate a new “Recording set” composed of the desired number of experimental “Conditions”. Each experimental “Condition” contains 12 or fewer signal acquisitions or trials.

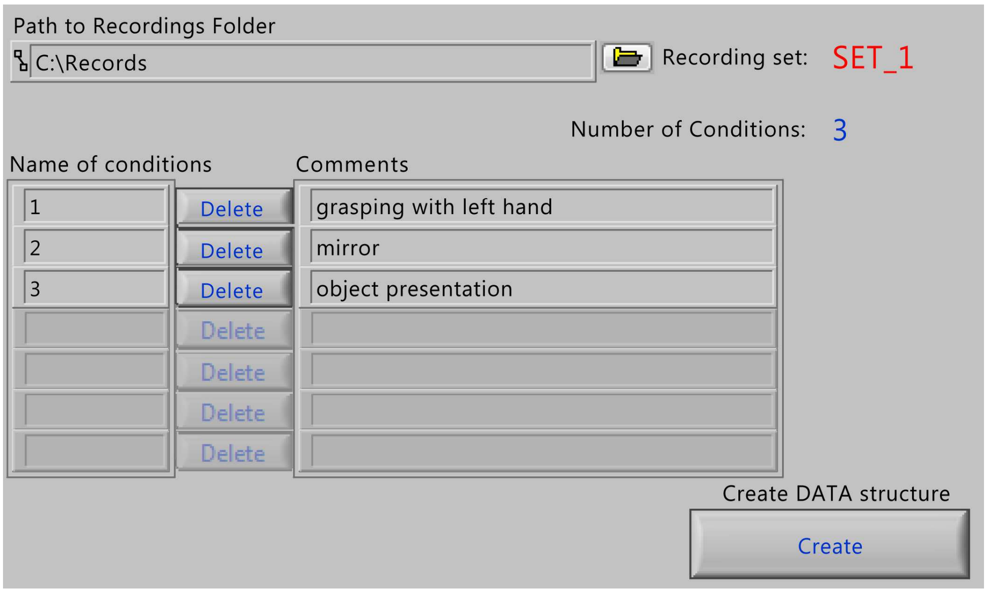

You may choose destination folder where your data folders will be created after pressing the button Create. Please remember to give meaningful names for “Conditions” since they are visualized during recordings.

Chapter 3.4 (Get instant Principal Components**)**

This interface window emerges after pressing the button Get PCs ("**14**" in Figure 3).

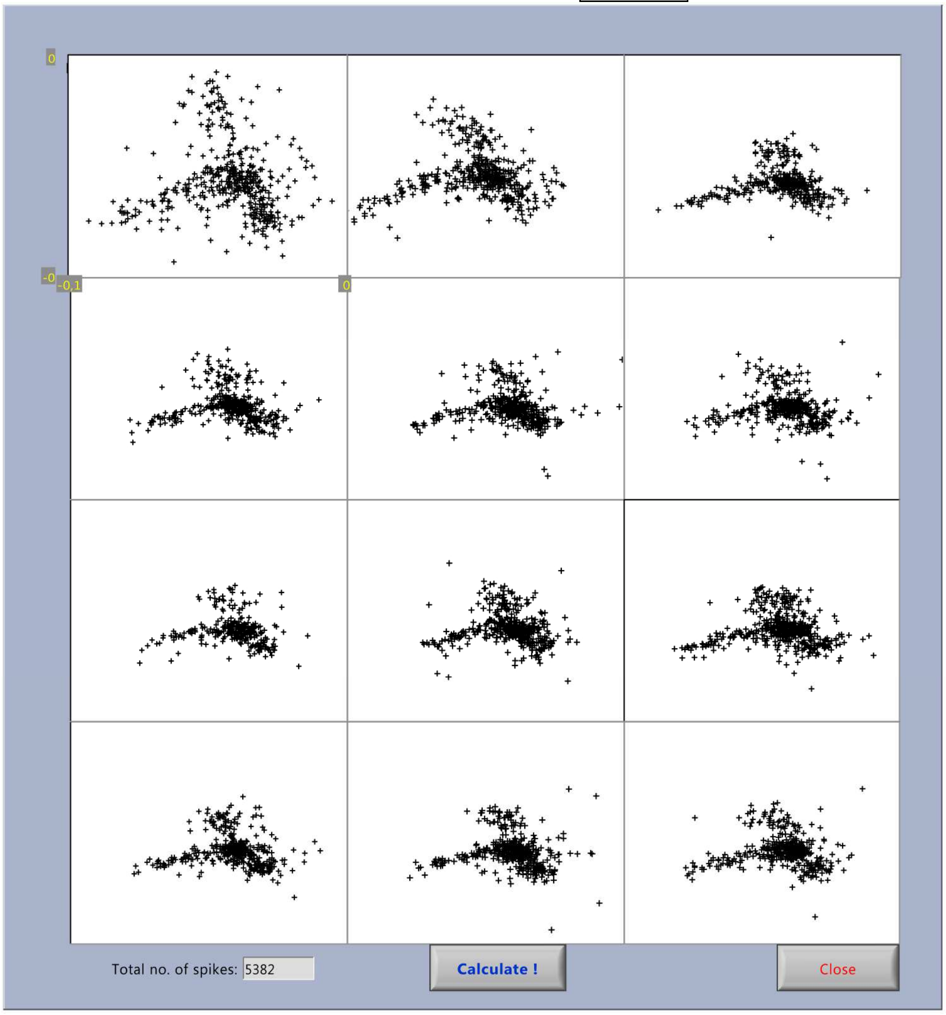

Scatter-plots of Principal Component 1 vs. Principal Component 2 for each spike waveform extracted automatically from the dataset. 2D spatial distribution of PCs gives the experimenter an instant view of the maths of the recorded dataset and the consistency of trials within the experimental "Condition".

Chapter 4. On-line Spike Sorting

/Figure 4/

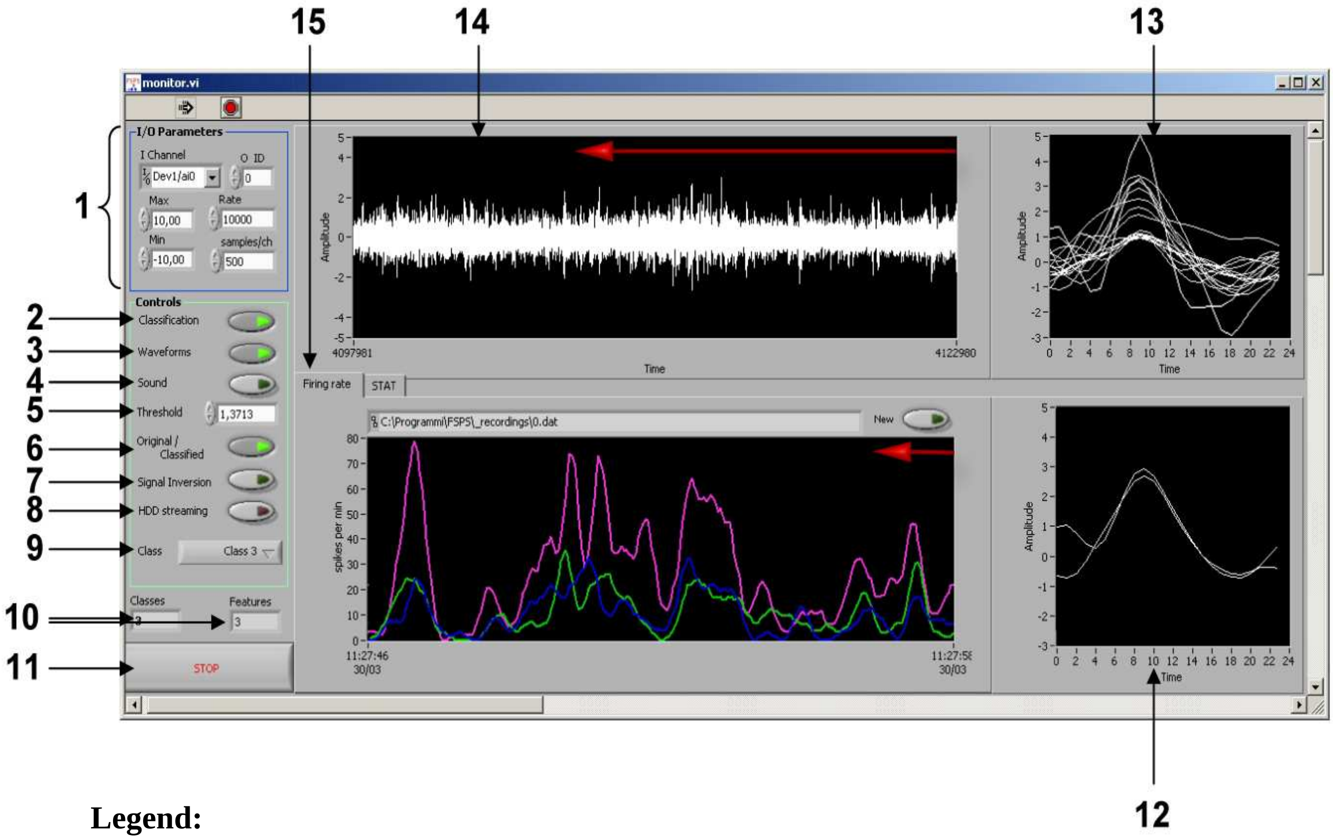

Legend:

1 - Control group for Input/Output parameters

2 - **Classification** control button to switch classification algorithm “ON” and “OFF” . *“ON” is default value.*

3 - **Waveform** control button to switch visualization of waveforms in indicators 12 and 13 “ON” and “OFF”. Switching “OFF” may slightly improve overall classification performance on some slow computers. *“ON” is default value.*

4 - **Sound** control button to switch “ON” and “OFF” sound generation and reproduction (both clicks or original signal, see “**6**”) through line-out of the computer board. *“OFF” is default value.*

5 - Actual threshold level for spike detection algorithm.*

6 - **Original/Classified** control button consents reproduction of "Original” signal picked up by electrode, or “Clicks” pertaining to classified waveforms though line-out of computer sound board. “*Classified*” *is default value.*

7 - **Signal Inversion** switches the inversion of the input signal “ON” and “OFF” .*

8 - **HDD streaming** switches “ON” and “OFF” data storage to HDD of the computer with a *filename*.dat, indicated above, as a Flow Chart indicating single unit activity.

9 - Pull down menu for selection of the isolated unit to be acoustically reproduced and visualized in “**12**”.

10 - Information panel displaying number of “Classes” and “Features” used.

11 **STOP** control button stops on-line monitoring and closes current window.

12 - Graph indicating spike shapes that successfully passed classification procedure and satisfied the parameters of class selected in “**9**”.

13 - Graph indicating all spike shapes extracted at current time instant.

14 - Moving chart indicator displaying raw signal.

15 - Flow chart displaying current firing rate of isolated and classified single units.

Note: * - settings are determined during “TEST” procedure (*offline*) and transferred from interface window shown in Figure 2.

Chapter 4.1 (Input/Output parameters)

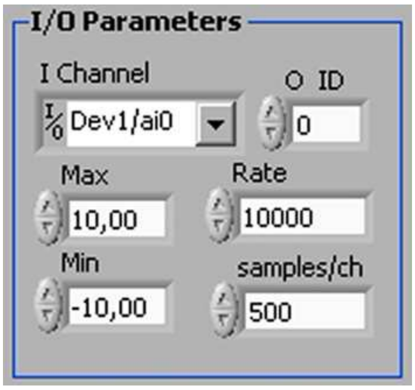

"I Channel" – The name and the number of DAQ Analogue Input channel connected to recording electrode. Its activity is visualized in the "Moving Chart" indicator (see Figure 4). By default, it is the same channel that has been used in "TEST" clustering procedure (Fig. 1a). *This parameter is set up automatically, but can be manually adjusted here if required.*

"Max" – maximal input voltage allowed (check your NI DAQ AI limits to set up this parameter). *This parameter is set up automatically, but can be manually adjusted here if required.*

"Min" – minimal input voltage allowed (check your NI DAQ AI limits to set up this parameter). *This parameter is set up automatically, but can be manually adjusted here if required.*

"O ID" – the number of computer sound boards in the system . *This parameter is set up automatically, but can be manually adjusted here if required.*

"Rate" –digitalization rate of the analogue input signal. It must be the same as that used during the "TEST" clustering procedure. *This parameter is set up automatically, but can be manually adjusted here if required (not recommended).*

"sample/ch"- the number of samples to be acquired during each measurement session. For instance, at a digitalization rate of 10,000 samples/s, the acquisition of 500 samples will take 20 ms *(default value)*. This can be adjusted to your specifications.

Chapter 4.2 (Flow Chart)

Firing rate Tab

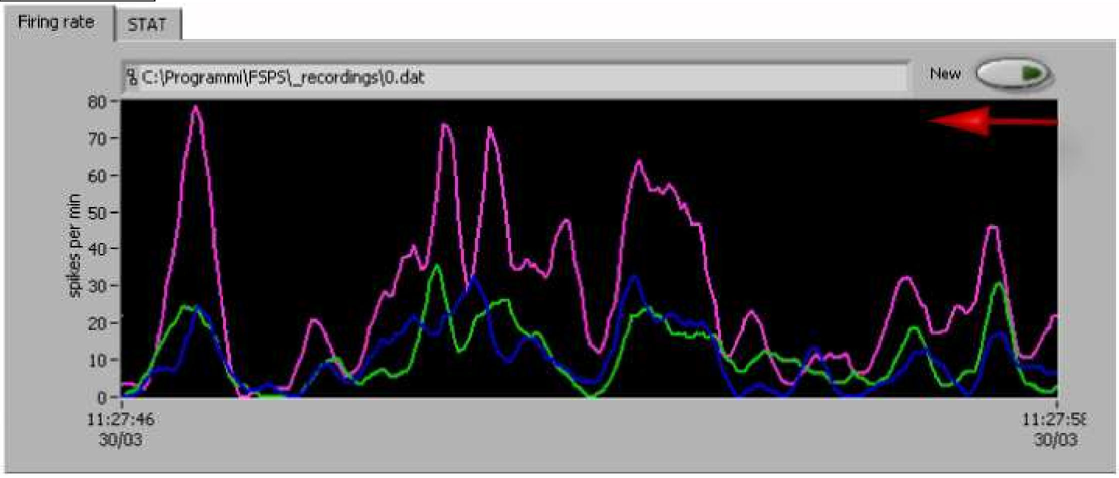

This Flow Chart shows the firing rate of isolated single units. At the top part of this Tab there is an indicator showing the file name and the path for data storage when the HDD streaming button is "ON". Pressing the button New (top right) creates a new empty file. The filenames will feature progressively increasing numbers (\1.dat, \2.dat, \3.dat, etc.).

STAT Tab

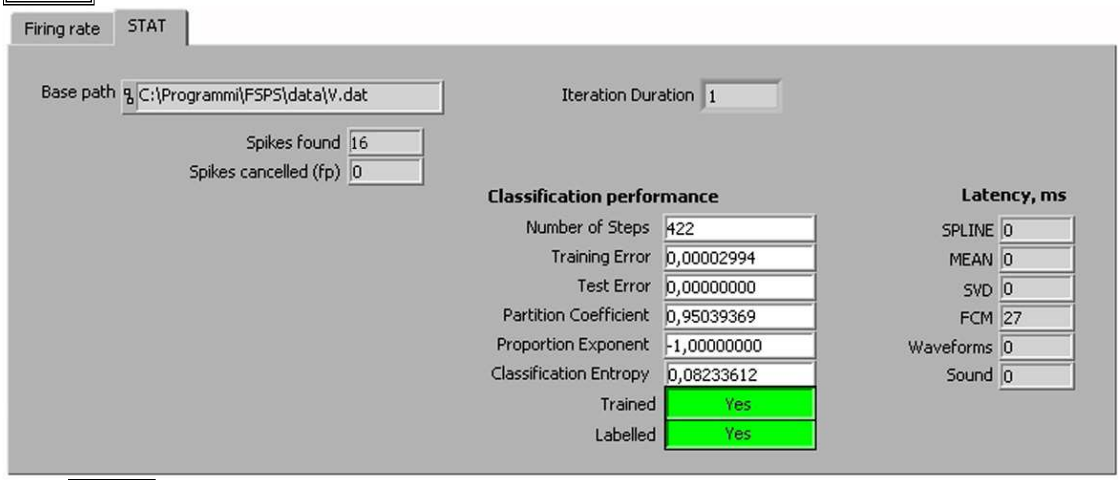

The STAT Tab shows some additional information about the on-line classification module performance .

- **"Base path"** shows the path for the V.dat file with the cluster prototype for your current recording. It is updated each time when you press the TEST button (see Chapter 2, Figure 2).
- **"Spikes found"** shows the number of spikes detected in the current time-frame and subjected to the automatic classification algorithm.
- **"Spikes cancelled"** shows the number of spikes in the current time-frame that have been cancelled by our false-positive-removing algorithm.
- **"Classification performance"** – group indicators showing basic Fuzzy C-Mean parameters and quality measurements.
- **"Latency, ms"** is a duration of each critical step of spikes classification in milliseconds.

Chapter 4.3 (HDD streaming)

While monitoring neuronal activity during experiments or clinical tests you may need to record some important fragments for future in-depth analysis. Pressing the button HDD streaming will start this process, saving the data in indicated file. To start a new file click the New button.

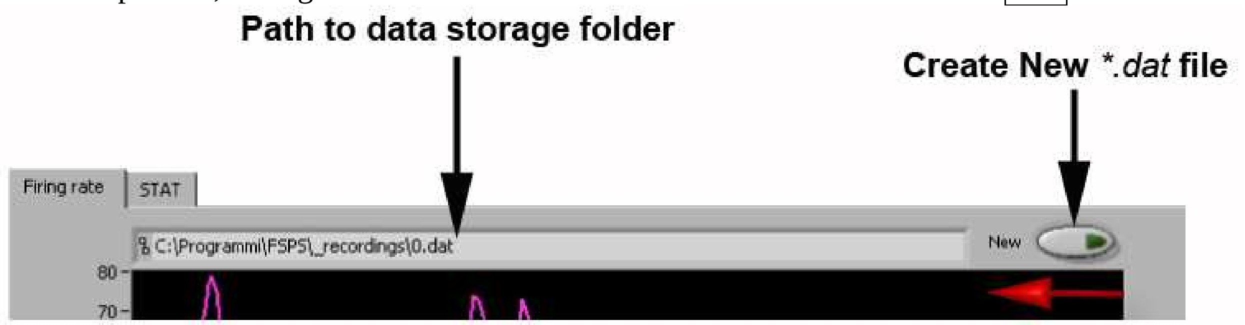

The number of your *.dat file will automatically increase.

You can select the source of your data to be saved depending using the Original/Classified (Figure 4) switch; "Original" saves raw signal data while "Classified" saves timestamps for isolated spikes only.

Examples and Supporting Files

Example files are located in the installation folder:
FE – contains triggered acquisition from the Monkey Premotor Cortex (area F5c) during goal-directed grasping movement. The acquisition is triggered at 1st second when the monkey touches the sensor. Experiments were performed at the University of Ferrara.
GE – this folder contains recordings of cortical neuronal discharges acquired by nanotubes at IIT, Genova. The signal is unfiltered, and activation of software filtering option is therefore necessary. Due to long recording you may need to process it in batches.
UD – several human recordings from internal GPi during Deep Brain Stimulation (Udine).
QUI – a text file (usually as “data.txt”) contains simulation data originally proposed and published by Q. Quiroga et al. (2004) (doi:10.1162/089976604774201631).

More recording to test the FSPS™ algorithm can be downloaded here: http://www.spikesorting.com/examples-demo.aspx

FAQ & Troubleshooting

Q: FSPS™ software does not run after installation. Why?

A:

a. This may happen on some computers running Windows 7 with particular “International settings”. To fix this, please, set English language for non-Unicode programs and/or apply English to the your “day & time” system format.
b. Run the programs as an Administrator, i.e. check “Administrator” box in the “Privilege level” menu (see Compatibility Tab of the FSPS.exe property).
c. Try to select another, more direct Programme folder during installation procedure, “C:\PSPS”, for example.

Q: FSPS™ software starts, but do not perform classification when I press “TEST” button. Thus, the only one class (blue spike shapes) is visualized.

A:

This may happen on some computers running 64-bit version of Windows. To make the classifier working correctly you should run FSPS™ software in the WindowsXP compatibility mode (see Compatibility Tab of the FSPS.exe property). However, we are working to make it compatible with all 64-bits operating systems.

Glossary

FCM - **Fuzzy C-Mean,** see *Tutorial* for details [http://home.dei.polimi.it/matteucc/Clustering/tutorial_html/cmeans.html]

FSPS™ - An abbreviation from **Fuzzy Spike Sorting,** see www.spikesorting.com

IIT - **Italian Institute of Technology** [http://www.iit.it]

PCA - **Principal Component Analysis,** see *here* [http://en.wikipedia.org/wiki/Principal_component_analysis] for details

PCs - **Principal Components**

PSVD - **Partial Singular Value Decomposition,** see *The Computer Journal (1987) 30 (3): 268-275. doi:10.1093/comjnl/30.3.268* for details

SVD - **Singular Value Decomposition,** see here [http://en.wikipedia.org/wiki/Singular_value_decomposition] for details

UNIFE - **University of Ferrara** [http://www.unife.it]
